# Plasmodesmata dynamics in bryophyte model organisms: secondary formation and developmental modifications of structure and function

**DOI:** 10.1007/s00425-024-04476-1

**Published:** 2024-07-04

**Authors:** Linus Wegner, Katrin Ehlers

**Affiliations:** Institute of Botany, Justus-Liebig University, 35392 Giessen, Germany

**Keywords:** *Anthoceros agrestis*, Development, Evolution, Intercellular transport, *Marchantia polymorpha*, *Physcomitrium patens*

## Abstract

**Main conclusion:**

Developing bryophytes differentially modify their plasmodesmata structure and function. Secondary plasmodesmata formation via twinning appears to be an ancestral trait. Plasmodesmata networks in hornwort sporophyte meristems resemble those of angiosperms.

**Abstract:**

All land-plant taxa use plasmodesmata (PD) cell connections for symplasmic communication. In angiosperm development, PD networks undergo an extensive remodeling by structural and functional PD modifications, and by postcytokinetic formation of additional secondary PD (secPD). Since comparable information on PD dynamics is scarce for the embryophyte sister groups, we investigated maturating tissues of *Anthoceros agrestis* (hornwort), *Physcomitrium patens* (moss*)*, and *Marchantia polymorpha* (liverwort). As in angiosperms, quantitative electron microscopy revealed secPD formation via twinning in gametophytes of all model bryophytes, which gives rise to laterally adjacent PD pairs or to complex branched PD. This finding suggests that PD twinning is an ancient evolutionary mechanism to adjust PD numbers during wall expansion. Moreover, all bryophyte gametophytes modify their existing PD via taxon-specific strategies resembling those of angiosperms. Development of type II-like PD morphotypes with enlarged diameters or formation of pit pairs might be required to maintain PD transport rates during wall thickening. Similar to angiosperm leaves, fluorescence redistribution after photobleaching revealed a considerable reduction of the PD permeability in maturating *P. patens* phyllids. In contrast to previous reports on monoplex meristems of bryophyte gametophytes with single initials, we observed targeted secPD formation in the multi-initial basal meristems of *A. agrestis* sporophytes. Their PD networks share typical features of multi-initial angiosperm meristems, which may hint at a putative homologous origin. We also discuss that monoplex and multi-initial meristems may require distinct types of PD networks, with or without secPD formation, to control maintenance of initial identity and positional signaling.

**Supplementary Information:**

The online version contains supplementary material available at 10.1007/s00425-024-04476-1.

## Introduction

Plasmodesmata (PD) are cytoplasmic nanochannels interconnecting plant cells to a dynamic symplasmic continuum. By mediating exchange of metabolites and signaling (macro)molecules, they control intercellular communication (Sager and Lee 2014; Li et al. 2021; Bayer and Benitez-Alfonso 2024). Thus, PD impact developmental coordination (Wu et al. 2016; Schreiber et al. 2024), metabolic adaptation (Tylewicz et al. 2018; Miras et al. 2022), and stress responses (Grison et al. 2019; Tabassum and Blilou 2022). PD presence in all extant land-plant taxa (embryophyta) emphasizes their essential role for complex multicellular plant organisms, which share ancestry with streptophyte algae (Raven 2005; Brunkard and Zambryski 2017; Wegner et al. 2023) and conquered land some 500 million years ago (de Vries and Archibald 2018; Bowman 2022). Most studies on PD have, however, been performed on angiosperms.

‘Canonical’ PD are composed of a plasma membrane-lined pore that traverses the cell wall and contains an ER-derived desmotubule (Nicolas et al. 2017). The membranes within PD are considered as microdomains with distinctive molecular compositions (Tilsner et al. 2016; Li et al. 2021; Béziat and Jaillais 2023). The narrow cytosolic sleeve between the desmotubule and the plasma membrane establishes the pathway for symplasmic exchange by diffusion or targeted transport (Crawford and Zambryski 2001). In type II PD morphotypes of mature tissues, the internal substructures are clearly visible, particularly in the dilated median PD regions. In contrast, the cytosolic sleeve can hardly be discerned in narrow type I PD of young tissues due to the tight attachment of plasma membrane and desmotubule. Counterintuitively, type I PD have a higher size exclusion limit (SEL), i.e., they mediate the diffusional transport of larger (macro)molecules (Nicolas et al. 2017).

To exert control on the size exclusion limit and on the rate of molecules trafficking through PD (Deinum et al. 2019; Peters et al. 2021), the width of the cytosolic sleeve may be regulated by MCTPs (multiple C2 domains and transmembrane region proteins) tethering the desmotubule to the plasma membrane (Li et al. 2021; Pérez-Sancho et al. 2023). Furthermore, deposition of callose controls the diameter of the PD orifices and often creates narrow neck constrictions to regulate PD permeability dynamically (Amsbury et al. 2018; Wu et al. 2018).

Different PD types have been distinguished based on their origin (Kragler et al. 1998; Ehlers and Kollmann 2001; Burch-Smith et al. 2011). Primary PD are generated during cell division by enclosing ER strands into the emerging cell plate (Hepler 1982; Li et al. 2023). Yet, additional secondary PD (secPD) appear postcytokinetically in existing walls to remodel the symplasmic networks in developing tissues, or to keep PD densities (PD/µm^2^) constant during massive expansion (Ehlers and Kollmann 2001; Ehlers and van Bel 2010).

Two modes of secPD formation have been described. De novo PD formation in locally degraded cell-wall areas (Kollmann and Glockmann 1991; Chambaud et al. 2022), establishes simple straight or branched secPD at (interspecific) interfaces, e.g., of grafts and host–parasite interactions (Fischer et al. 2021; Kurotani and Notaguchi 2021). Alternatively, during tissue development, PD twinning produces secPD (strands) which develop from pre-existing template PD to generate laterally adjacent PD pairs (Seagull 1983; Faulkner et al. 2008; Ehlers and van Bel 2010). Y-, X-, and H-shaped PD represent intermediates of the rapid twinning process that may increase PD numbers two- to threefold within 48 h (Fitzgibbon et al. 2013). Multiple twinning events may result in incomplete separation or fusion of neighboring PD strands, and give rise to complex branched PD with multiple orifices at either side, which are linked by a dilated central cavity (Faulkner et al. 2008; Ehlers and Große-Westerloh 2013; Fitzgibbon et al. 2013). During the sink–source transition of developing leaves, conversion of simple into complex branched PD coincides with a reduction of the size exclusion limit (Oparka et al. 1999; Crawford and Zambryski 2001).

Among non-seed plants, an increasing number of model plants has been established (for bryophytes: Szövényi et al. 2015; Bowman et al. 2017, 2022; Li et al. 2020; Rensing et al. 2020; Naramoto et al. 2022; Bi et al. 2024). Moreover, there are initial attempts to identify evolutionary conserved PD components by (phylo)genomics and (phylo)proteomics (Table S1 for bryophytes). However, only few studies have reported on structural PD modifications in non-seed plants and often focused on specialized tissues like food and water conducting cells (Table S2 for bryophytes). Even less studies have investigated functional PD properties in these taxa and predominantly investigated protonema filaments of the moss *Physcomitrium patens* (Table S1 for bryophytes).

Yet, PD densities have been determined by counting PD in the apical meristems of various seed and non-seed plants. Two distinct types of PD networks were detected which strictly correspond to the meristem type (Imaichi and Hiratsuka 2007; Mansouri 2012; Imaichi et al. 2018; Fig. S1). Simplex and duplex meristems, e.g., of seed-plant sporophytes, possess multiple initials and exhibit interface-specific PD networks (IPD), where low PD densities are kept constant at all interfaces via secPD formation. Contrarily, lineage-specific PD networks (LPD) occur in monoplex meristems, e.g., of fern sporophytes and bryophyte gametophytes. Here, highly abundant PD mark the interfaces of the single initials, but PD densities successively decline during development of the derivatives, which was attributed to a lack of secPD formation (Imaichi and Hiratsuka 2007; Mansouri 2012; Imaichi et al. 2018). It has even been hypothesized that the evolution of different meristem types with their respective PD networks had been driven by evolutionary gains or losses of the general ability to form secPD (Evkaikina et al. 2014, 2017; Fig. S1). Following this rationale, ferns and bryophytes should completely lack the capability to form secPD. However, a few reports, indicating the occurrence of secPD in bryophytes, cast doubt on the hypothesis (Schnepf and Sych 1983; Table S1).

In the present study, we systematically investigated whether bryophyte gametophytes form secPD. By transmission electron microscopy (TEM) on three model organisms, representing hornworts, liverworts, and mosses, we compared PD networks during tissue development and found quantitative evidence for secPD formation via (incomplete) PD twinning in all major bryophyte taxa. We conclude that PD twinning is an ancient evolutionary trait that was likely present in the most recent common ancestor of bryophytes (and possibly embryophytes). Thus, absence (or insignificance) of secPD formation in the monoplex gametophyte meristems of bryophytes (Fig. S1, Table S1) seems to be targeted and may function in the maintenance of the initial’s identity and in positional signaling. For our initial study on the PD network of a bryophyte sporophyte, we chose the basal meristem of the model hornwort, which is special due to its indeterminate growth with multiple initials (Renzaglia et al. 2008; Frangedakis et al. 2021, 2023). Remarkably, we observed targeted secPD formation and an IPD, resembling that of seed plants. Finally, our TEM analyses revealed additional taxon-specific structural PD modifications in the maturating bryophyte gametophytes (Table S2), which resemble those of angiosperms, e.g., transition of type I into type II-like PD and pit-pair formation. We discuss their putative function in wall-thickening growth. Using fluorescence redistribution after photobleaching (FRAP), we demonstrated that the PD permeability becomes drastically reduced in maturating phyllids of the moss model, as observed for angiosperm leaves.

## Materials and methods

### Plant material

The bryophyte model plants *Anthoceros agrestis*, *Physcomitrium patens*, and *Marchantia polymorpha* were obtained from the MAdLand consortium of the DFG priority program 2237. *A. agrestis* (‘Saxony’ formerly ‘Bonn’) was grown under axenic conditions on solid Knop minimal medium under long-day conditions (16/8 h at 70–90 µmol*m^−2^ s^−1^) at 22 °C (Frangedakis et al. 2021, https://www.hornworts.uzh.ch/en/hornwort-protocols.html). Axenic *A. agrestis* cultures with sporophytes were provided by PD Dr. P. Szövényi, University of Zurich, Switzerland. *M. polymorpha* (TAK-1) and *P. patens* (Reute) were grown in the greenhouse under high humidity, at 20–24 °C and 16/8 long-day conditions with automated additional SON-T illumination. A *P. patens* (Reute) marker strain, constitutively expressing mCherry (Perroud et al. 2019), was obtained from Prof. Dr. A. Hiltbrunner, University of Freiburg, Germany. Stereomicroscopy of living plants (Fig. S2a) was performed with a Leica M165 C (Leica Microsystems, Wetzlar, Germany), equipped with a Leica DFC 450 camera driven by the Leica Application Suite software (Version 4.3.0).

### Light microscopy and TEM

Sample preparation for light and electron microscopy followed Althoff et al. (2022) with slight adaptations (Methods S1). Semithin sections of 0.5–1 µm thickness, cut with glass knifes on a Reichert Om U2 ultramicrotome (Leica Microsystems GmbH, Wetzlar, Germany), were stained with 0.5% crystal violet and observed under a Leica DM 5500 microscope B equipped with the above-mentioned camera for sample selection (Fig. S2g–l). Ultrathin sections (~ 80 nm) were cut with a diamond knife, transferred onto single-slot copper grids coated with formvar films, and contrasted with 2% uranyl acetate and lead citrate (Reynolds 1963) for 12 min each. Sections were analyzed with an EM912AB TEM (Zeiss, Oberkochen, Germany) at 120 kV accelerating voltage under zero-loss energy filtering conditions. Micrographs were recorded with a 2 k × 2 k dual-speed slow-scan CCD camera (SharpEye, TRS, Moorenweis, Germany) using the iTEM software package (OSIS). Figure plates were mounted with Corel PHOTO-PAINT (2021, Version 23.1.0.389, Corel, Ottawa, Canada) and measurements were performed with ImageJ (1.53e; Wayne Rasband and contributors National Institutes of Health, USA).

### Sample selection for PD counts

For PD counts on bryophyte gametophytes, we differentiated between mature tissues and young tissues, showing onset of cell vacuolation and development of intercellular spaces. Sample selection of cross-sectioned young and mature *P. patens* phyllids and thallus regions of *A. agrestis* and *M. polymorpha* was performed via light-microscopy analyses (Fig. S2).

PD were counted in anticlinal walls between upper epidermal cells using three biological replicates for each species and developmental stage. At least 30 (and up to 108; Table S3) walls were analyzed per replicate. To obtain these numbers, serial sections were used in some replicates. Using mature thalli of *A. agrestis*, we exemplarily proved that results of PD counts and calculations of derived parameters did not differ significantly between serial sections (Methods S2).

For PD counts in *A. agrestis* sporophytes, three biological replicates per developmental stage were longitudinally sectioned. The small initial cells analyzed in the basal meristem were separated from the larger differentiating cells selected from a more apical sporophyte region by a distance of 50–150 µm (Fig. 5b). For each replicate, PD were counted in 32–50 anticlinal walls, oriented orthogonal to the growth axis, and 36–52 periclinal walls running parallel to the growth axis (Table S4). Analyzed walls were selected from the peripheral tissue region giving rise to the future assimilative parenchyma.

### PD counts

For calculations of PD densities and frequencies, we counted only those PD strands which could unambiguously be identified under the TEM and clearly extended into the electron-opaque cell-wall layers. Both, simple and branched PD were considered as single PD. In addition, the number of orifices per PD was counted separately on each wall half to estimate the degree of PD branching, and to calculate orifice densities and frequencies (Ehlers and van Bel 2010). PD occurring within a maximal lateral distance of 200 nm from each other were characterized as twinned PD. Furthermore, PD were classified as type II(-like) PD if they exhibited visible cytosolic sleeves and desmotubules (Nicolas et al. 2017).

### Calculations and statistics

For each biological replicate, we compiled (Tables S3, S4, S5):

(i) the total numbers of PD and orifices observed,
(ii) the mean outer PD radius,
(iii) the total numbers and shares of branched, twinned, and type II(-like) PD,
(iv) the total analyzed cell-wall length determined from TEM overview images, and
(v) the wall thickness at the PD position or, for mature *A. agrestis* gametophytes, next to the pit pairs.

PD densities (PD/µm^2^ cell wall, Tables S3, S4) were computed from the total PD number and the total cell-wall length analyzed per replicate considering the mean outer PD radius and the section thickness (Gunning 1978; Methods S3). PD density values of individual walls were calculated, respectively, and their distributions were depicted in violin plots to compare developmental stages (Fig. S3).

To estimate PD frequencies for each replicate (total number of PD/average cell-interface area; Tables S3, S4), we computed the respective interface areas (Table S5; Ehlers and van Bel 2010). The mean length of the anticlinal epidermal walls analyzed in TEM cross sections represents the average cell height, which was multiplied with the average cell(-wall) length determined from top view light-microscopy images for each species and developmental stage. In replicates of *A. agrestis* sporophytes, the average cell-wall length of periclinal walls calculated from the longitudinal TEM sections was complemented by light-microscopic measurements on tangential walls in cross sections. Anticlinal cell-wall areas were directly measured in cross sections (Table S5).

Per replicate, we also calculated the sum of squares of the orifice numbers found in either wall half to consider the three-dimensional PD profile (Ehlers and van Bel 2010). Dividing this value by the sum of PD observed in either wall half delivers the average degree of PD branching. Multiplying this factor with the PD densities or frequencies resulted in the orifice densities or frequencies, respectively (Tables S3, S4).

For statistical analysis, means and standard deviations were calculated for the investigated characters of each set of replicates. The two different developmental stages per species were usually compared by two-sample Student’s *t*-tests (R version 4.3.0 using RStudio 2023.06.1 + 524 "Mountain Hydrangea"). *F*-tests for variance homogeneity were used, and Welch’s tests and/or log-transformation were applied for comparison if this pretest was negative. Shapiro–Wilk tests for normal distribution were positive, except for a few cases where the investigated character was absent in one or both developmental stages (0% values, e.g., Fig. 4q). Kruskal–Wallis test was used for the comparison of wall thickness of *A. agrestis* (Fig. S4a) with pairwise Wilcoxon Rank Sum tests as post-hoc test.

### FRAP

For successful FRAP experiments (Wróbel-Marek et al. 2022), whole *P. patens* wildtype gametophores were stained with 1 mM 5-(and-6)-carboxyfluorescein diacetate (CFDA) for 30 min at RT, washed four times in tap water, and incubated in tap water overnight in the dark to guarantee an uniform tissue staining. Mature, intermediate, and young phyllids were carefully dissected from *P. patens* mutants expressing mCherry, embedded in 2% low-gelling agarose type VII (Sigma-Aldrich, Steinheim, Germany) on slides, and stored overnight in the dark under high humidity levels to recover from wounding stress. Observation and documentation were performed with a Leica TCS SP8 confocal laser scanning microscope (Leica, Bensheim, Germany) using the Leica Application Suite X software (Version 3.5.7.23225). CFDA was excited by a 488 nm argon laser and mCherry by a diode pumped solid state 561 laser at 561 nm. Emission was detected at 507–537 nm and 590–630 nm, respectively, via PMT detectors using 40 × or 63 × immersion objectives (HCX PL APO CS2 40x/1.10 WATER and HCX PL APO CS2 63 × /1.20 WATER). Whole phyllid cells were bleached at 50–80% argon laser power and with 100% intensity of the 476 nm, 488 nm and 496 nm laser lines for 7–60 frames (0.648 s per frame, 512 × 512 px, 400 Hz bidirectional scan, 2 AU pinhole, ‘Zoom In’ setting). For mCherry bleaching, the 561 nm laser line was added. Images were assembled in short movies (Movies S1, S2) using Clipchamp (Version 2.9.1.0, Microsoft, Redmond, USA).

For graphical presentation, all FRAP data were corrected for photobleaching during the post-bleach image acquisition with a factor determined for a surrounding, unbleached region (pre-bleach intensity divided by the post-bleach intensity at the same time point). FRAP data were also normalized by setting the pre-bleach intensity of the target cell to the value 1 (100%; Kappel and Eils 2004).

## Results

Bryophytes lack orthologs of typical angiosperm PD proteins used in fluorescence-based live-imaging approaches to study PD development (Fitzgibbon et al. 2013; Brault et al. 2019; Table S1). Thus, we chose TEM analyses to characterize the developmental modifications of PD networks in gametophytes of the model organisms *Anthoceros agrestis* (hornwort), *Physcomitrium patens* (moss), and *Marchantia polymorpha* (liverwort). Being aware that modifications of PD networks often occur during tissue differentiation, we counted PD in largely comparable tissues in a young, but non-meristematic, and a mature stage. We focused on the anticlinal walls in *P. patens* phyllids and between upper epidermis cells of the two thalloid bryophytes, respectively (Fig. S2). Quantitative variables were calculated from the data to track the developmental alterations of PD numbers (Tables S3, S4). Further, we monitored additional modifications of the PD structure and studied functional alterations using FRAP experiments. Analyses were complemented by TEM studies on the basal meristem of *A. agrestis* sporophytes, where we compared PD between the multiple initials with those between differentiating derivatives.

### SecPD formation by twinning is adjusted to wall expansion in *Anthoceros agrestis* and pits develop around type I PD during wall thickening

**Fig. 1:**
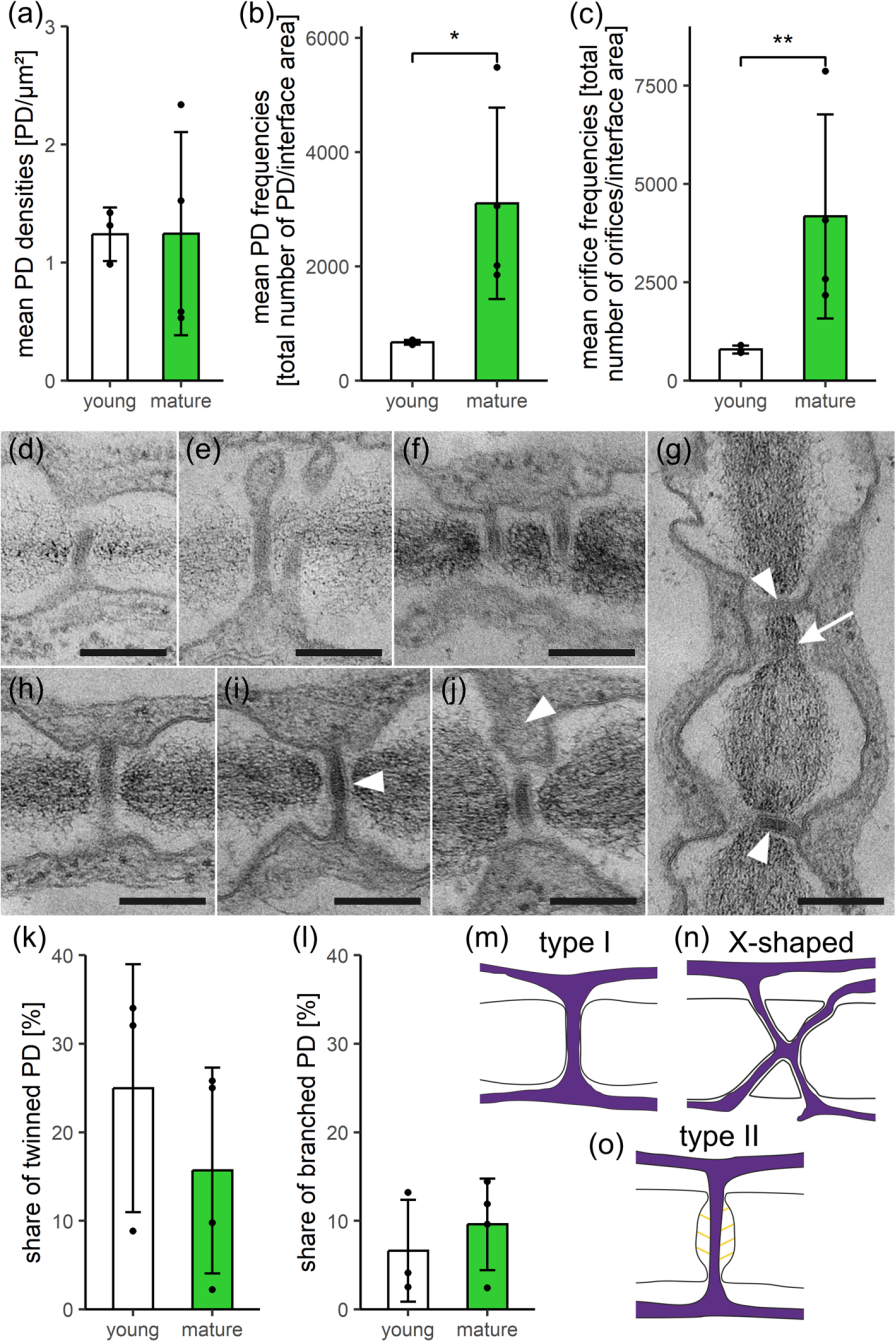
Comparison of quantitative PD data and PD ultrastructure in young and mature *Anthoceros agrestis* gametophytes. **a** Constant PD densities (PD/µm^2^ cell wall) concomitant with uniform developmental increases in (**b**) PD frequencies (total number of PD/average cell-interface area) and "Developmental increase" pertains to both frequency factors (**c**) orifice frequencies (total number of orifices/average cell-interface area) indicated formation of predominantly simple secPD during cell growth. TEM images of young (**d**, **e**) and mature walls (**f–j**), both showing single (**d**, **h**–**j**) and laterally adjacent pairs of twinned PD (**e**–**g**). Presumably, a multiple PD twinning event is captured in (**g**), where the first twinned PD pair had already been separated during wall expansion (arrowheads), and the derived upper PD had served as template for the second twinning event forming another secPD (arrow; peripherally sectioned). Electron-lucent wall collars were more prominent with mature PD (**f**–**j**, arrowhead in **i**). In thicker areas of mature walls, PD were located in short ‘pit membranes’ traversing narrow pit pairs (**g**, **i**, **j**, arrowhead in **j**). Similar shares of (**k**) twinned and (**l**) (mostly X-shaped) branched PD in young and mature walls likely indicated continuous secPD formation by twinning. **m–o** Simplified schematic drawings of PD morphotypes. **m** Narrow, simple type I PD, in which inner substructures could hardly be discriminated, often occurred in *A. agrestis* (**d**-**j**).** n** X-shaped branched PD might represent an intermediate of PD twinning. **o** Type II PD morphotypes with clear cytosolic sleeves were not found in *A. agrestis*. Graphs in (**a**–**c**, **k**, **l**) show means, standard deviations and data points of three young and four mature biological replicates, with a total of 129 and 194 cell walls analyzed, respectively. Significance levels: *p < 0.05, **p < 0.01. Original data shown in Table S3. Scale bars in (**d**–**j**): 200 nm.

Between upper epidermal cells of *A. agrestis* gametophytes*,* PD densities (PD/µm^2^ cell wall) were low and remained constant in young and mature thallus regions (means and standard deviations in Table S3; Fig. 1a), although cell growth caused a massive 5.2-fold increase of the average cell-interface area (from approx. 550 to 2800 µm^2^; Table S3). To balance PD densities during wall expansion, additional secPD must have been inserted consistently, as indicated by similar distribution patterns of PD densities in individual walls of both developmental stages (Fig. S3a). Computations of PD frequencies (total number of PD/average interface area) showed a significant 4.6-fold increase of PD numbers during tissue development (Fig. 1b) which seemed to be roughly adjusted to the degree of wall expansion. A similar significant 5.3-fold increase in orifice frequencies (total number of orifices/average cell-interface area; Fig. 1c) indicated that the degree of branching did not differ between the two developmental stages (1.2 vs. 1.3 orifices/PD half; Table S3). Thus, secPD resembled the primary PD with their predominantly simple, unbranched morphology.

High numbers of secPD between epidermal cells most likely developed from PD twinning. Besides single simple PD (Fig. 1d, h–j, m), closely adjacent PD pairs were regularly found without significant quantitative differences in young and mature samples (Fig. 1e–g, k). Sometimes the spatial arrangement of neighboring PD hinted at multiple successive twinning events (Fig. 1g). Further, a low, but steady percentage of the analyzed PD was branched in both, young and mature tissues (Fig. 1l, n), and had an X-shaped morphology typical for intermediates of the PD twinning process (Faulkner et al. 2008). Branched PD with three orifices on the same side were rarely found with only 6% of the branched PD.

Altogether, these observations could best be explained by an ongoing multiple PD twinning process throughout epidermal growth in *A. agrestis* gametophytes, as observed with angiosperm tissues (Fitzgibbon et al. 2013). Sites of local wall degradation, which would be a typical indication for de novo secPD formation (Chambaud et al. 2022), were not observed in the young samples. In mature tissues, PD were often located in the short ‘pit membranes’ of narrow pit pairs (Fig. 1g, i, j), where the wall was only half as thick as in the surrounding region (121 vs. 259 nm; Fig. S4a). Possibly, the pit pairs, which lacked considerable thickening growth, may have aided the PD twinning process, as PD pairs regularly occurred in the pit membranes (Fig. 1g). Yet, PD also traversed non-pitted wall regions, which were likewise significantly thinner than the walls around pit pairs (188 vs. 259 nm; Fig. S4a). This suggests that there might be a general threshold of approx. 200 nm wall thickness that requires the onset of pit formation. All PD of *A. agrestis* epidermal cells had a type I PD morphology (Fig. 1d–j, m, Table S3). They did not exhibit visible cytosolic sleeves or desmotubules (Fig. 1o), and kept a narrow inner diameter of 23–27 nm throughout development (Nicolas et al. 2017). Yet, electron-lucent wall collars predominantly occurred with mature PD.

### SecPD formation by twinning is not adjusted to wall expansion in *Physcomitrium patens* and type I PD transform into type II-like morphotypes with different functional properties

**Fig. 2:**
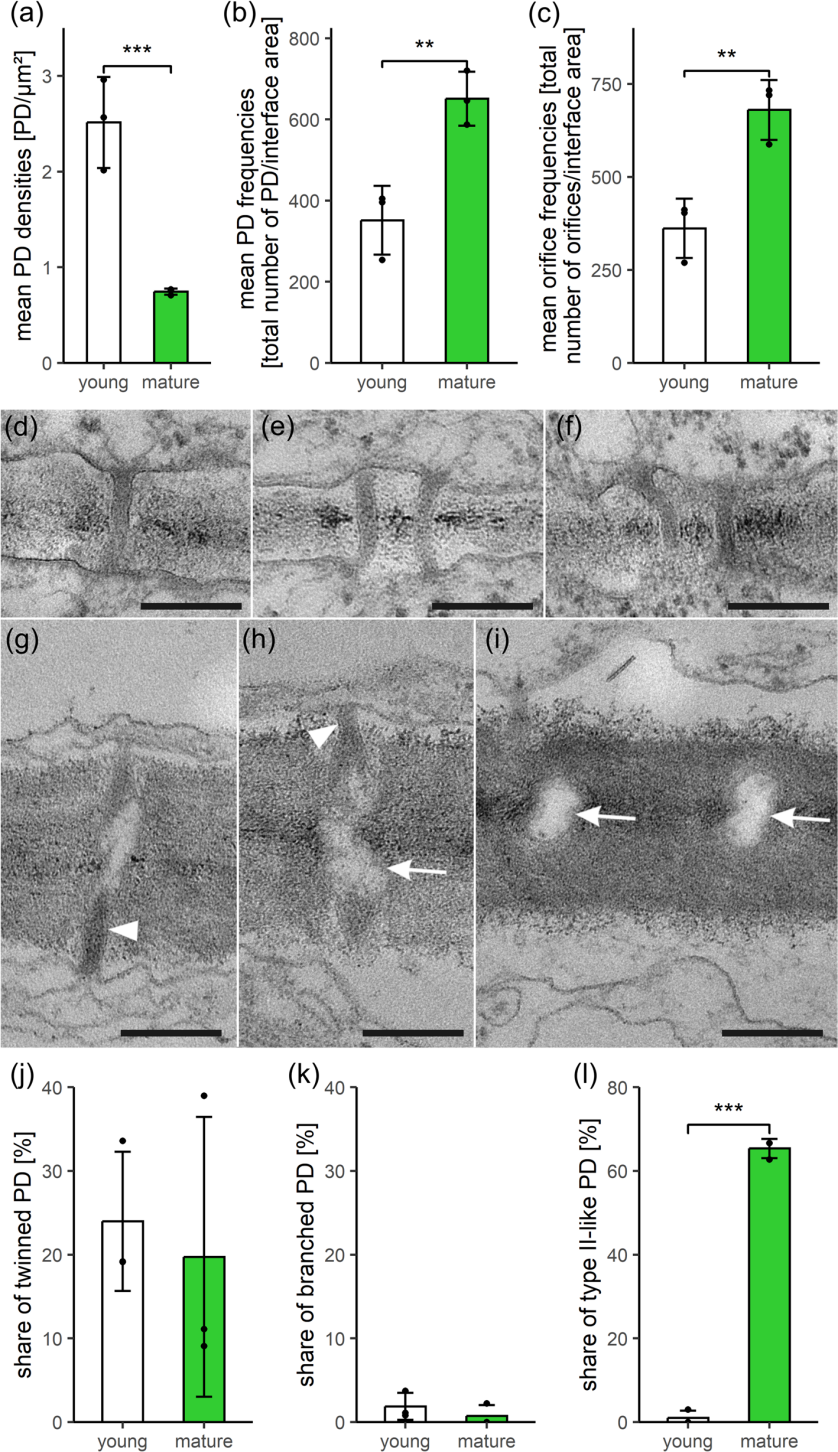
Comparison of quantitative PD data and PD ultrastructure in young and mature phyllids of *Physcomitrium patens* gametophytes. **a** Despite decreasing PD densities, **b** PD frequencies and (**c**) orifice frequencies increased uniformly during tissue development, indicating formation of simple secPD. TEM images of young (**d–f**) and mature walls (**g–i**), both showing single (**d**, **g**, **h**) and laterally adjacent, twinned PD (**e**, **f**). Neighboring PD in (**i**) were not counted as twinned PD, because they were separated too far from each other. Similar shares of (**j**) twinned and (**k**) (mostly Y-shaped) branched PD in young and mature walls suggested secPD formation by twinning throughout development. **l** Young, thin walls were traversed by narrow type I PD with no discernible inner substructures (**d**–**f**). During tissue maturation and wall thickening, the majority of PD had been transformed into type II-like PD (**g**–**i**) with constricted neck regions of variable lengths (arrowheads in **g**, **h**) and conspicuous median dilatations (arrows in **h**, **i**), containing cytosolic sleeves and bloated desmotubules. Graphs in (**a**–**c**, **j**–**l**) show means, standard deviations, and data points of three biological replicates each, with a total of 205 and 148 cell walls analyzed, respectively. Significance levels: **p < 0.01, ***p < 0.001. Original data shown in Table S3. Scale bars in (**d**–**i**): 200 nm.

Contrary to *A. agrestis*, low PD densities in the anticlinal walls of the single-cell layer constituting phyllids of *P. patens* gametophytes decreased significantly during tissue development (3.4-fold; Fig. 2a), coinciding with a massive 6.3-fold increase of the average cell-interface area (from approx. 140 to 875 µm^2^; Table S3). Consequently, distribution patterns of PD densities changed toward abundant low-density walls in mature phyllids (Fig. S3b). Nevertheless, both, PD and orifice frequencies increased significantly to almost double values in mature tissues (1.9-fold each; Fig. 2b, c). This proved secPD formation in developing *P. patens* phyllids, and, since the degree of PD branching did not change (both 1.0 orifices/PD half; Table S3), primary and secPD typically had a simple, unbranched morphology. Yet, the numbers of secPD were obviously not sufficient to balance the dilution of pre-existing PD during cell (wall) expansion, resulting in the overall decline of PD densities.

Moderate secPD formation in *P. patens* phyllids can most probably also be attributed to PD twinning, with constant ratios of single (Fig. 2d, g, h, i) and twinned PD (Fig. 2e, f, j) in both developmental stages, like in *A.agrestis*. The steady portion of branched PD with a Y-shaped morphology in *P. patens* phyllids (Fig. 2k) was, however, even lower than in the hornwort tissues. This might be explained by the lower PD twinning activity in the moss phyllids resulting in the observation of only few branched intermediates, and formation of only few secPD.

Locally degraded wall areas, indicative for de novo secPD formation, were never observed with *P. patens* phyllids, and special wall collars as well as pit pairs were lacking, although the mature walls reached a considerable thickness (579 nm; Fig. S4b). In these thick walls, the majority of PD exhibited a pronounced type II morphology with striking median dilatations (Fig. 2g–i, l). However, these PD are termed ‘type II-like’, since their desmotubules were also enlarged and partially impaired the visibility of the cytosolic sleeve (Nicolas et al. 2017; Fig. 2g–i). In the thinner walls of younger tissues (159 nm; Fig. S4b), type II-like PD with moderate dilatations were only rarely observed (Fig. 2l). This suggests a developmental type I-to-type II-like transformation correlated to wall-thickening growth.

**Fig. 3:**
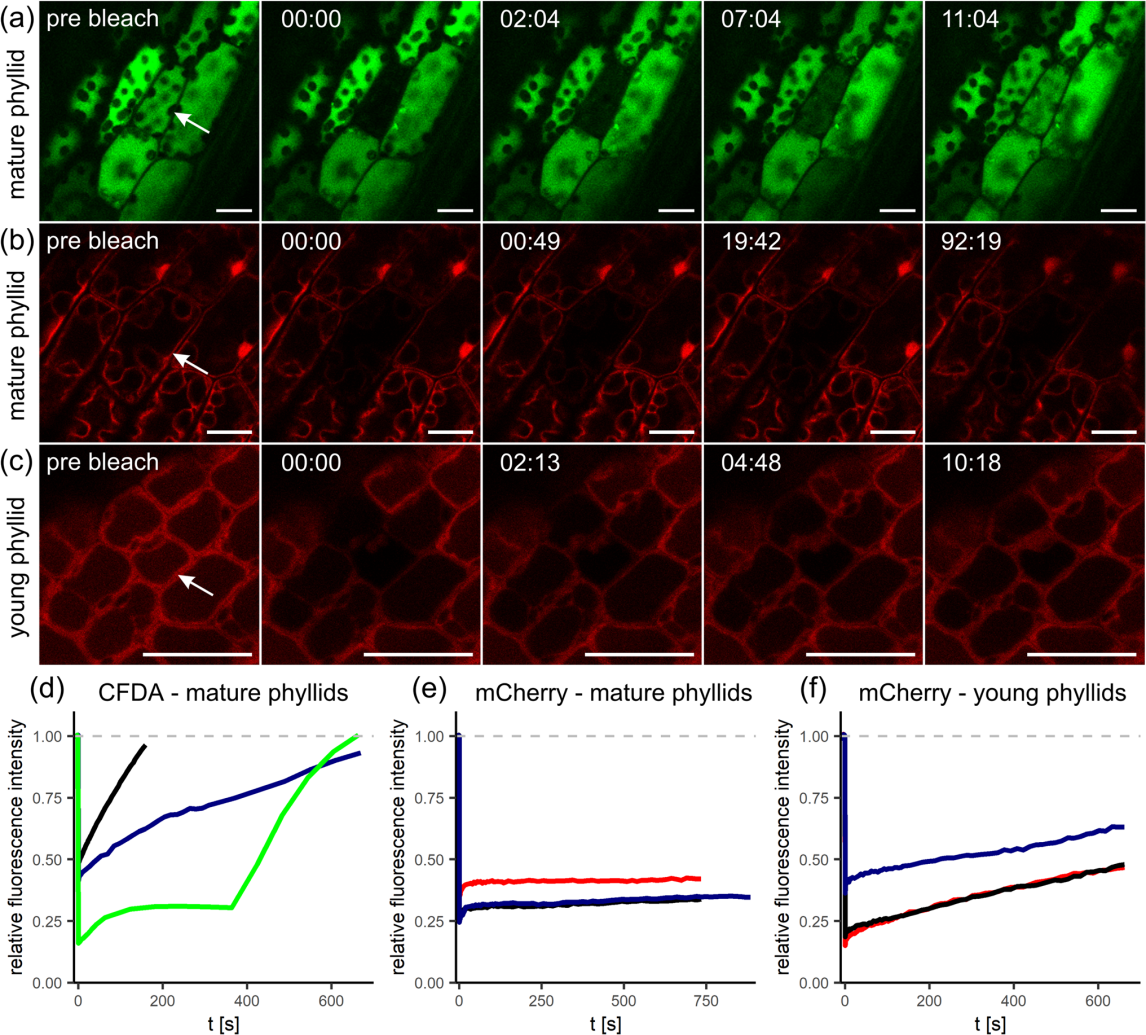
Fluorescence redistribution after photobleaching (FRAP) in *P. patens* phyllids over time (min:s). **a** Bleached cell (marked with arrows throughout) in a mature wildtype phyllid showing rapid fluorescence redistribution due to symplasmic transfer of the small CF(DA) (376 Da) from the neighboring cells. **b** In contrast, no PD transport of the larger mCherry (26.7 kDa) was observed in mature phyllids of a *P. patens* mCherry marker line. **c** In young phyllids of the same line, mCherry fluorescence redistribution occurred rapidly after bleaching. **d–f** Progression of FRAP over time shown for three representative repetitions of each experimental approach, starting with the pre-bleach intensity of the target cell (100%). The green and red graphs correspond to the experiments shown in (**a**–**c**), respectively. The depicted normalized, relative fluorescence intensities often show a fast, but weak, initial fluorescence redistribution within the first seconds, which might originate from other focus planes of the bleached cell. Thereafter, a continuously increasing fluorescence intensity indicated symplasmic transfer from adjacent cells within 10–20 min. Further details in Table S6, Movies S1, S2. Scale bars: 20 µm

The functional capacities of simple type I and type II-like PD morphotypes in *P. patens* phyllids were tested with FRAP experiments using the fluorescent tracers carboxyfluorescein (CF, 376 Da, derived from CF-diacetate) and mCherry (26.7 kDa). Redistribution of CF(DA) fluorescence occurred within 10–20 min in 79% of the experiments performed on mature wildtype phyllids (*n* = 14; Fig. 3a, d, Table S6, Movie S1). This indicated rapid symplasmic transport of the small dye from the neighboring cells into the bleached cell through type II-like PD. However, the considerably larger mCherry, constitutively expressed in a *P. patens* marker strain (Perroud et al. 2019), was never transported within mature phyllids (*n* = 13; Fig. 3b, e, Table S6), even when observation times were prolonged up to 105 min. In contrast, mCherry moved cell-to-cell in the youngest accessible phyllids, resembling the young developmental stage used for TEM analyses, which could be assumed to still possess type I PD. Here, rapid fluorescence redistribution was observed with 83% of the experiments (n = 6; Fig. 3c, f, Table S6, Movie S2), whereas with slightly older phyllids in an intermediary stage, this percentage dropped to 22% (n = 9; Table S6). Obviously, functional PD capacities of developing *P. patens* phyllids were gradually modified, resulting in a downregulation of the size exclusion limit and/or the rates of non-targeted transport of larger molecules, coinciding with the transition of simple type I into simple type II-like PD.

### PD numbers do not increase in *Marchantia polymorpha*, but (complex) branched PD presumably develop via incomplete twinning and type I PD transform into type II-like morphotypes

**Fig. 4:**
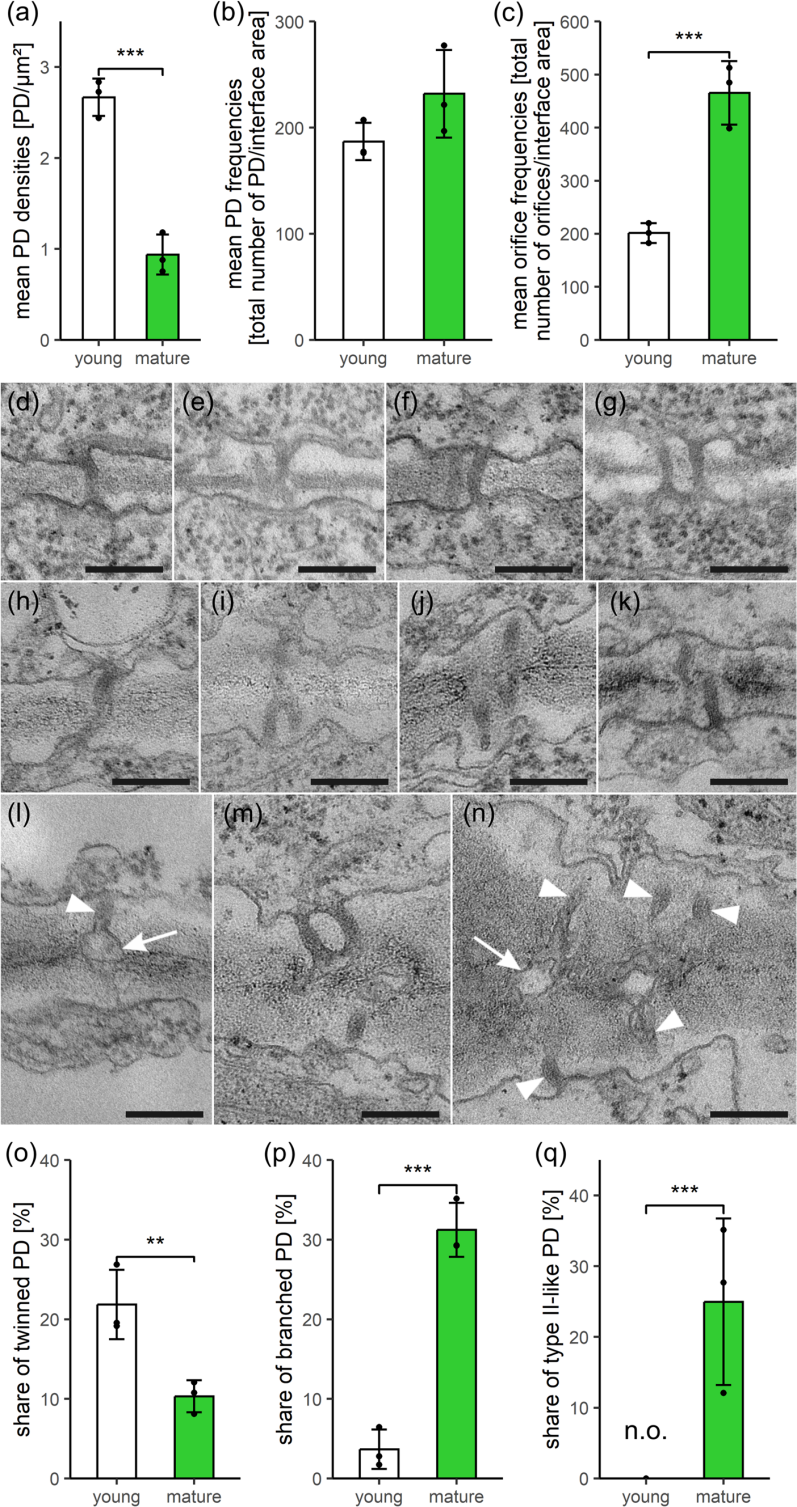
Comparison of quantitative PD data and PD ultra-structure in young and mature *Marchantia polymorpha* gametophytes. **a** Although PD densities decreased and **b** PD frequencies remained constant in the moderately growing walls, **c** orifice frequencies raised to double values. This indicated the addition of new orifices to the existing PD, giving rise to (complex) branched PD. TEM images of young (**d–g**) and mature (**h–n**) walls, showing simple PD (**d**, **h**, **l**) next to twinned PD pairs (**f**, **g**), and Y-, X-, or H-shaped branched PD, regarded as intermediates of twinning (**e**, **i**, **j**, **k**), as well as complex branched PD with multiple orifices (**m**, **n**). **o** Decreasing shares of twinned PD and **p** concomitant increasing shares of (complex) branched PD in developing walls suggest that (complex) branched PD might have arisen from incomplete PD twinning. Branched PD formation also coincides with (**q**) the emergence of type II-like PD in mature walls (**l**–**n**) with constricted neck regions (arrowheads in **l**, **n**) and median dilatations/central cavities (arrows in **l**, **n**) containing visible cytosolic sleeves and dilated desmotubules. Graphs in (**a**–**c**, **o**–**q**) show means, standard deviations, and data points of three biological replicates each, with a total of 309 and 246 cell walls analyzed, respectively. n.o.: not observed. Significance levels: **p < 0.01, ***p < 0.001. Original data shown in Table S3. Scale bars in (**d**–**n**): 200 nm.

Like in *P. patens* phyllids, low PD densities between upper epidermal cells of *M. polymorpha* gametophytes decreased significantly during development (2.8-fold; Fig. 4a), while the cell-interface area increased moderately (3.6-fold, from approx. 70 to 250 µm^2^; Table S3). The distribution patterns of PD densities also changed toward abundant low-density walls in mature thalli (Fig. S3c). Yet, *M. polymorpha* differed from the other bryophytes, because PD frequencies remained stable throughout development (Fig. 4b). Anyhow, orifice frequencies raised significantly to more than the double value at maturity (2.3-fold; Fig. 4c), concomitant with an increasing degree of PD branching (1.1 vs. 2.0 orifices/PD half; Table S3, Fig. S5a). Thus, rather than producing separate secPD by twinning or de novo formation, *M. polymorpha* obviously added new orifices to the existing PD to generate (complex) branched morphotypes. However, the number of newly formed PD strands was apparently not adequate to compensate for the PD dilution during wall expansion and, consequently, orifice densities still decreased significantly (1.5-fold, 2.9 vs. 1.9 orifices/µm^2^; Table S3, Fig. S5b), but not to the same extent as the PD densities.

The formation of (complex) branched PD from simple ones has often been observed during angiosperm development (Oparka et al. 1999; Fitzgibbon et al. 2013), but the mechanism of this transformation has been a matter of debate (Ehlers and Kollmann 2001; Faulkner et al. 2008; Burch-Smith et al. 2011). In view of the constant PD frequencies, it appears unlikely that branched PD morphotypes in *M. polymorpha* arose from fusion of laterally neighboring PD. Instead, (complex) branched PD morphotypes most probably can be attributed to incomplete PD twinning (Faulkner et al. 2008; Ehlers and Große-Westerloh 2013). Simple (Fig. 4d, h, l), (complex) branched (Fig. 4e, i, j, k, m, n), and twinned PD profiles (Fig. 4f, g) were observed in both developmental stages, and branched PD often exhibited the Y-, X-, or H-shaped morphology typical for intermediates of PD twinning (Figure j, k). Yet, complex branched PD with three or more orifices on the same wall side were regularly observed with 16% of the branched PD. In contrast to the other bryophytes, ratios of twinned and (complex) branched PD were not constant throughout development (Fig. 4o, p). Data of young *M. polymorpha* tissues resemble those of *A. agrestis* and *P. patens*, but, in mature liverwort samples, the share of twinned PD decreased significantly (2.1-fold; Fig. 4o), while the ratio of (complex) branched PD increased massively (8.5-fold; Fig. 4p). This coincidence suggests that the moderate wall growth of *M. polymorpha* was not sufficient to properly separate PD pairs during twinning events. Due to ‘incomplete’ twinning, twinned PD profiles would only seldom be formed and ‘intermediate’ branched PD morphotypes would accumulate and eventually give rise to complex branched PD in the mature tissues. Spatial control of wall expansion might support this process (Ligrone and Duckett 1998; Faulkner et al. 2008).

Pit formation was not observed in the *M. polymorpha* epidermal walls, but a quarter of the mature PD exhibited a type II-like morphology with clear median dilatations/central cavities where enlarged desmotubules hampered the visibility of the cytosolic sleeve (Fig. 4l–n, q). They must have developed from type I PD representing the only morphotype in young walls (Fig. 4q). Remarkably, the type II-like morphology was more frequently found with (complex) branched PD than with simple PD (56 vs. 14%; Fig. S6) indicating a direct correlation between the two characters. Again, the type II-like morphology coincided with the thickness of the mature walls, which was only 216 nm in the vicinity of type I PD, but 342 nm around type II-like PD (Fig. S4c).

### The basal meristem of *Anthoceros agrestis* sporophytes exhibits an interface-specific PD network and targeted formation of secPD occurs during development of the cell derivatives

**Fig. 5:**
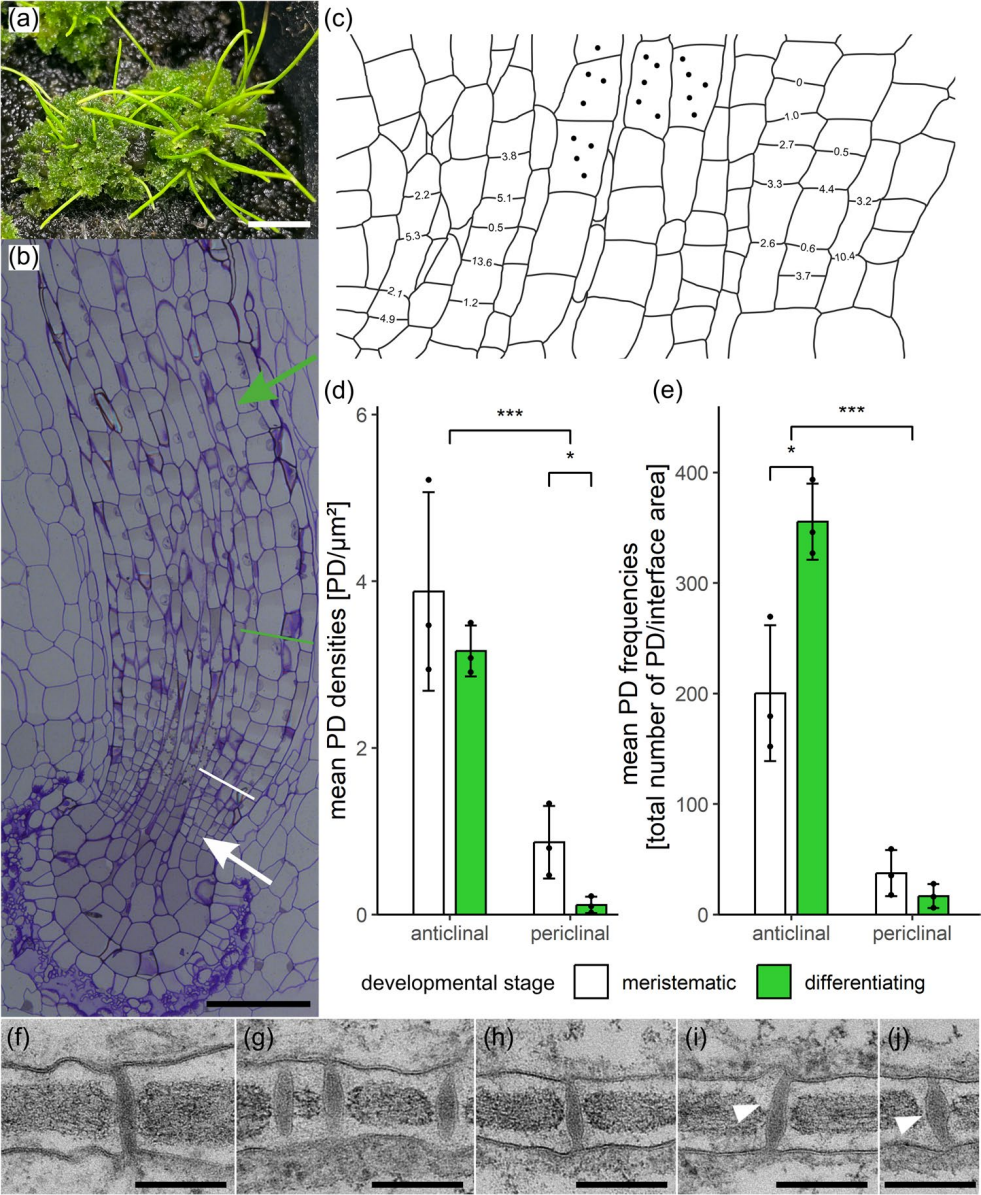
Quantitative PD data and PD ultrastructure of the multi-initial basal meristem of *Anthoceros agrestis* sporophytes and its differentiating derivatives. **a** Sporophytes growing on *A. agrestis* gametophytes. **b** Light micrograph of a longitudinally sectioned sporophyte. The meristematic zone (white arrow) with the basal initials is connected to the differentiating peripheral assimilative parenchyma (green arrow) via longitudinal cell files (between white and green line). **c** Spatial distribution of PD densities, exemplarily indicated for individual anticlinal walls of a basal meristem (averaged from two serial sections), exhibited no regular pattern. Dots mark the central columella cells whose cell files were excluded from the PD counts. **d** PD densities were generally lower in the periclinal cell walls and decreased further during development, while anticlinal walls maintained constant PD densities. Concomitantly, **e** low, but constant PD frequencies were found in periclinal walls, whereas PD frequencies of anticlinal walls were significantly higher and increased during development, indicating targeted secPD formation. **f**–**j** TEM images of type I-like PD in walls of differentiating cells, surrounded by prominent electron-lucent wall collars (arrowheads in **i**, **j**). Internal substructures could hardly be discerned, but the outer PD shape showed slightly enlarged median regions. Graphs in (**d**, **e**) show means, standard deviations, and data points of three biological replicates each, with a total of 108 and 143 anticlinal walls, as well as 119 and 133 periclinal walls analyzed, respectively. Significance levels: *p < 0.05, ***p < 0.001. Original data shown in Table S4. Scale bars in (**a**) 5 mm, (**b**) 100 µm, (**f**–**j**) 200 nm.

Having shown that all three bryophyte taxa produce secPD (strands) in developing gametophytes, we chose the basal meristem of *A. agrestis* sporophytes (Fig. 5a, b) for an initial quantitative study on PD networks in bryophyte sporophyte meristems. PD were counted in anticlinal and periclinal walls of the meristematic zone and between differentiating derivatives (Fig. 5b) giving rise to the future assimilative tissue.

The spatial distribution of PD densities in individual walls of the basal meristem (Fig. 5c) showed no regular pattern of declining densities, contrary to a LPD (Fig. S1). Moreover, PD densities were low in both anticlinal and periclinal walls (Fig. 5d), with the overall average of 2.6 PD/µm^2^ (Table S4) being within the typical range of tracheophyte meristems with an IPD (Fig. S1). Low maximum PD densities of individual *A. agrestis* walls (17.2 PD/µm^2^; Fig. S3d, e), and a high number of walls without observed PD, also matched with the typical features of tracheophyte IPDs (Fig. S1; Imaichi and Hiratsuka 2007; Imaichi et al. 2018). The PD network in the multi-initial basal meristem of *A. agrestis* sporophytes can, therefore, be classified as IPD and, thus, differs clearly from the LPDs found in gametophyte meristems of liverworts and mosses with single initials (Mansouri 2012; Ligrone and Duckett 1998; Table S1). To our knowledge, no quantitative investigations of PD networks have been performed so far with gametophyte meristems of hornworts.

Villarreal Aguilar (2006; Villarreal Aguilar and Renzaglia 2006) mentioned abundant PD in a gametophytic and a basal sporophytic meristem of two hornwort species, and a decline during cell differentiation*,* which has not been quantitatively corroborated, though. Throughout development of *A. agrestis* sporophyte tissues, significantly lower PD densities and PD frequencies occurred in periclinal walls between cell files than in anticlinal walls within cell files (Fig. 5d, e). Moreover, in developing periclinal walls, the decreasing PD densities (7.5-fold; Fig. 5d), resulting in absence of observed PD with the majority of differentiating walls (Fig. S3e), and concomitant constant PD frequencies (Fig. 5e) suggest lack of secPD formation. In anticlinal walls, however, PD densities as well as their distribution patterns remained constant (Figs. 5d, S3d), and PD frequencies raised 1.8-fold (Fig. 5e). This indicates targeted formation of secPD adjusted to wall expansion, exclusively in anticlinal walls.

Although hardly any branched PD were observed in the anticlinal walls (both stages 0.2%; Table S4), similar shares of twinned PD throughout development (14 vs. 12%; Table S4, Fig. 5g) favor twinning as the mode of secPD formation. In contrast to the *A. agrestis* gametophyte, no pit formation occurred in the relatively thin walls of differentiating sporophyte cells (≤ 230 nm). Yet, PD often had slightly enlarged median regions of up to 45.5 nm inner diameter, but they did not resemble type II PD since the cytosolic sleeve and the desmotubule could still never be identified (Fig. 5f–j; Nicolas et al. 2017). Instead of an alteration of the inner PD (sub)structure, the prominent electron-lucent wall collar surrounding the PD (Fig. 5f–j) might have contributed to the modification of only the outer PD shape.

## Discussion

### Taxon-specific variability of PD structure and function in bryophytes

PD densities of the analyzed model bryophyte tissues were low (0.1–3.9 PD/µm^2^; Tables S3, S4), but comparable to those observed in spermatophytes (Seagull 1983; Evert et al. 1996; Zhu et al. 1998a; Fuchs et al. 2011; Deng et al. 2023). PD densities in protonema filaments of *P. patens* were much higher (11.3 PD/µm^2^; Gombos et al. 2023), and PD morphology differed from that of phyllids (Table S2, Fig. 2g–j), suggesting that PD networks of the juvenile and adult growth forms of moss gametophytes are not alike, which is also supported by functional studies (Table S1). Rapid cell-to-cell transport of dendra2 (26.1 kDa) has been observed in protonema filaments of *P. patens*, while, in accordance with our results, the dye moved only very slowly in mature phyllids (Kitagawa and Fujita 2015; Table S1). Possibly, intercellular exchange via vesicles might also play a physiologically significant role in bryophytes, since vesicular structures occurred with strikingly high abundance in close vicinity of cell walls (Wu and Gallagher 2014; Cui et al. 2020; Ruf et al. 2022; Fig. S7), but a detailed analysis was beyond the scope of this study.

Our quantitative data showed that analyses of PD densities (PD/µm^2^) cannot always deliver a complete picture of PD networks, but data must be considered in relation to cell (wall) growth (PD frequencies) and the degree of PD branching (Fig. 4a–c, p). In particular, declining PD densities cannot be taken as definitive indication for a lack of secPD, but may simply reflect low rates of secPD formation which cannot compensate for the PD dilution in expanding walls (Fig. 2a, b; Ehlers and van Bel 1999).

**Fig. 6:**
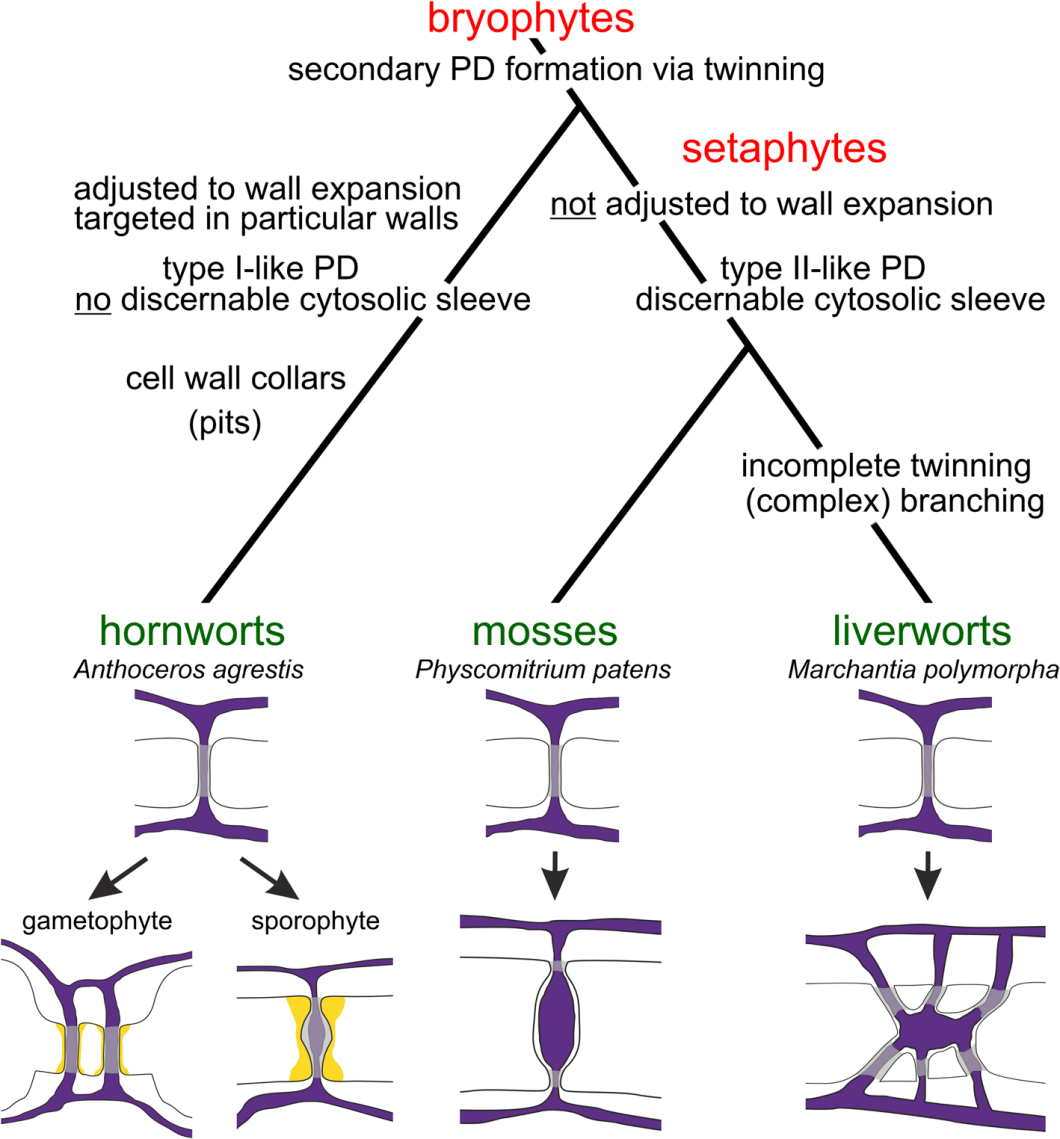
Schematic drawing summarizing significant alterations observed with PD networks in developing tissues of the major bryophyte taxa, which match the recent phylogeny (Bechteler et al. 2023). PD development started from simple type I PD in all taxa (schematic drawings upper row). Schematic drawings below depict the major PD features in *A. agrestis* mature gametophytes (narrow pit pairs and wall collars around simple type I PD), *A. agrestis* differentiating sporophytes (wall collars, constricted neck regions, and slight median dilatations of type I-like PD), *P. patens* mature gametophytes (simple type II-like PD with median dilatations containing visible cytosolic sleeves and dilated desmotubules), and *M. polymorpha* mature gametophytes (complex branched type II-like PD with median cavities containing visible cytosolic sleeves and dilated desmotubules). Gray overlay marks PD regions in which no internal (sub)structures could be discerned. Yellow: wall collars, violet: ER/desmotubules

With our comprehensive data set, we detected secPD formation in all bryophyte taxa, which most likely takes place via (incomplete) PD twinning. This gives rise to twinned PD pairs in *A. agrestis* and *P. paten*s, and to (complex) branched PD with newly added orifices in *M. polymorpha* (Fig. 6). There are only few other reports indicating the occurrence of secPD in non-meristematic tissues of bryophytes which support our results (Table S1). PD counts on moss phyllids of *Sphagnum palustre* (Schnepf and Sych 1983) proved formation of simple secPD, comparable to those observed with *P. patens* in this study (Figs. 2, 6). Branched PD, resembling those observed with *M. polymorpha* (Figs. 4, 6), have previously been found in distinct liverwort species (Table S1). At that time, the branched PD morphology was regarded as typical character of de novo formed secPD (Ding et al. 1992, 1999; but see Ehlers and Kollmann 2001; Burch-Smith et al. 2011), and (incomplete) PD twinning had not yet been discovered.

With respect to the well-supported monophyletic origin of the three major bryophyte taxa (Wickett et al. 2014; Puttick et al. 2018; de Sousa et al. 2019), it can now be supposed that secPD formation via twinning is an ancient evolutionary trait that was already present in the most recent common ancestor of bryophytes (Fig. 6). It cannot be answered as yet, whether the origin of secPD formation can even be attributed to the most recent common ancestor of embryophytes and independent losses of the trait occurred, e.g., in ferns. Alternatively, secPD formation could have evolved independently in bryophytes and tracheophytes (Fig. S1 for scenarios). Quantitative investigations on PD networks in non-meristematic tissues of other non-seed plants might deliver further insights. Remarkably, secPD formation has already been reported for the streptophyte algae *Chara corallina* of the distantly related embryophyte sister group Charophyceae (Franceschi et al. 1994), but this trait might have originated from convergent evolution similar to that of phaeophycean algae (Brunkard and Zambryski 2017; Wegner et al. 2023).

SecPD formation in *A. agrestis* was tightly controlled. It might be absent in periclinal sporophyte walls (Fig. 5d, e), but mostly, it was precisely adjusted to cell (wall) expansion (Figs. 1a, b, 5d, e, 6). Such a strict correlation was not observed with the members of the monophyletic setaphyte clade (mosses and liverworts), where formation of secPD (orifices) lagged behind wall expansion (Figs. 2a, 4a, S5b, 6). Instead, both setaphytes showed a developmental transformation of narrow type I PD into dilated type II-like PD with visible cytosolic sleeves and enlarged desmotubules (Figs. 2l, 4q, 6). Descriptions of PD with median dilatations in mature tissues of various setaphyte species (Table S2) support this observation, and may hint at a flexible and differentially controlled adjustment of their PD networks. The type I-to-type II(-like) transition might be required to keep sufficiently high symplasmic transport rates in walls exceeding 200 nm thickness. Modeling predicted that larger cytosolic sleeves would be favored in thicker walls to increase PD permeation efficiency (Deinum et al. 2019). Yet, transport rates of (complex) branched type II-like PD with ‘clustered’ orifices, as found with *M. polymorpha*, may differ from evenly distributed simple type II-like PD, observed with *P. patens*, despite similar orifices frequencies (Deinum et al. 2019; Peters et al. 2021; Bayer and Benitez-Alfonso 2024).

Concomitant with the morphological transition, the size exclusion limit controlling non-targeted PD transport was restricted in developing *P. patens* phyllids (Fig. 3), resembling previous reports for angiosperm leaves during the sink-to-source transition (Oparka et al. 1999; Crawford and Zambryski 2001; Fitzgibbon et al. 2013). Possibly, this functional alteration can be attributed to changes in the narrow necks of the type II(-like) PD, which are regarded as bottlenecks for symplasmic transport. They are controlled by diverse mechanisms, including actin, callose and MCTP tether proteins (White et al. 1994; Amsbury et al. 2018; Pérez-Sancho et al. 2023; Renzaglia et al. 2024). Interestingly, beside enzymes regulating callose deposition occurring in PD proteomes of both setaphytes, MCTPs were identified as components of *P. patens* PD and it would be interesting to investigate, whether they are also present and functional in *M. polymorpha* (Table S1).

Consistent with previous observations on hornworts (Table S2), PD of *A. agrestis* kept their type I(-like) morphology throughout development (Figs. 1, 5, 6, Tables S3, S4), although in particular the sporophyte PD had slightly enlarged outer shapes within conspicuous wall collars (Figs. 5f–j, 6; Table S2; Paterlini et al. 2022; Bayer and Benitez-Alfonso 2024). Supposedly, hornwort PD lack particular molecular components mediating the developmental transition into type II(-like) PD, and PD (sphingo)lipid composition as well as MCTPs might be interesting candidates (Grison et al. 2015; Brault et al. 2019; Yan et al. 2019; Li et al. 2021; Pérez-Sancho et al. 2023). Transport rate limitations, which can be anticipated during wall thickening of *A. agrestis* (Deinum et al. 2019), may be counterbalanced by higher rates of secPD formation (Figs. 1a, 5d, 6). Slight dilatations of the PD diameter and pit-pair formation around type I PD in thicker walls (Figs. 1, 5, 6) may also counteract the functional challenges. Yet, pit pairs might not be an exclusive character of hornworts, but have also been reported for setaphyte species (Table S2).

The large variety of morphological and functional bryophyte PD modifications, which presumably rely on their respective molecular machinery, resembles that of angiosperm PD. This is remarkable, since, according to initial studies (Table S1), the molecular composition of bryophyte and angiosperm PD differs significantly, and important regulatory proteins are missing in the bryophyte PD proteomes (PDLPs, PDCBs, CALS/GSLs; Table S1). When selecting bryophyte material for molecular analyses, it should be considered that characters and molecular components of PD in distinct tissues might not be alike (Table S2, Fig. 6). This has already been demonstrated for the protein composition of type I and type II PD in *Arabidopsis thaliana* (Brault et al. 2019).

### PD networks in bryophytes meristems

Our study demonstrates formation of additional secPD (strands) in maturating gametophytes of all major bryophyte taxa and—in a targeted manner—also in the IPD of the multi-initial sporophytic meristem of *A. agrestis*. Consequently, the question arises, why secPD formation has not been detected in the monoplex meristems of setaphyte gametophytes with LPDs (Mansouri 2012). The previous hypo-thesis that a general inability to form secPD had evolved in these taxa (Evkaikina et al. 2014, 2017; Fig. S1) can now definitely be ruled out. In monoplex meristems of other land-plant taxa, branched putative intermediates of PD twinning were occasionally observed (Imaichi and Hiratsuka 2007; Evkaikina et al. 2017; Imaichi et al. 2018, Fig. 3.28 in Mansouri 2012). This possibly indicates secPD insertion, which however is insufficient to keep PD densities constant in the apices with single initials and LPDs.

It might also be hypothesized that setaphytes avoid (massive) secPD formation in their gametophytic apices to establish a gradient of PD densities from the initial to the successive derivatives, which meets the functional requirements of this meristem type. This interpretation would contribute to the discussion whether monoplex meristems of the gametophyte-dominant bryophytes represent the ancestral state or resulted from reductive evolution (Donoghue et al. 2021; Fouracre and Harrison 2022; Frangedakis et al. 2023).

High(est) abundance of PD at the interfaces between the single apical cell and its latest derivatives within LPDs of monoplex apices (Mansouri 2012) may help to maintain the stem cell identity of the pluripotent initial and its indeterminate growth, including control of mitotic activity and patterning of cell divisions. Further, it may be required to establish gradients of morphogenic signals including those required for apical dominance (Suzuki et al. 2021; Thelander et al. 2022; Beveridge et al. 2023; Flores-Sandoval et al. 2024).

It has been supposed that the determinate growth of *Azolla pinnata* fern roots is due to a progressive decrease of PD densities during successive apical cell divisions, causing an increasing symplasmic isolation of the initial, which then terminates mitotic activity (Gunning 1978). Auxin/cytokinin signaling may play a role in this system (de Vries et al. 2016). In angiosperms, PD densities decrease similarly with root age in *A. thaliana* (Zhu et al. 1998b) or in the dormant cambium of *Populus nigra* (Fuchs et al. 2010). Moreover, during the developmental switch from 2-D protonema to 3-D gametophore growth of the fern *Onoclea sensibilis*, the positioning of the initial’s oblique division wall coincided with the PD density distribution and could be predicted prior to its formation (Tilney et al. 1990). In the dormant *P. nigra* cambium, high PD densities likewise indicate the position of the first cell division in spring (Fuchs et al. 2010). It might be promising to investigate whether similar mechanisms also control meristem activity and determinate growth of the short-lived apical initial of moss sporophytes and/or of the intercalary meristems of setaphyte sporophytes (Fouracre and Harrison 2022; Flores-Sandoval et al. 2024).

IPDs within simplex or duplex meristems interconnect the multiple initials by uniformly low PD numbers (Zhu et al. 1998a; Imaichi and Hiratsuka 2007; Imaichi et al. 2018), which might support equality and interchangeability of the initials, allowing a more plastic developmental fate (van den Berg et al. 1995). This is regulated by tightly controlled signaling over small distances including PD-mediated exchange, as exemplified by the WUSCHEL/CLAVATA pathway (Yadav et al. 2011; Somssich et al. 2016; Kitagawa and Jackson 2017). Targeted secPD formation outside of the proper initial zone may possibly be decisive for initiating the differential development of the derivatives via symplasmic exchange of morphogens and positional information, which determine cell fates and cell identities (Furuta et al. 2012; Kitagawa and Jackson 2017; Linh and Scarpella 2022; Schreiber et al. 2024). Interestingly, low PD densities interconnect the initials in *A. thaliana* root meristems, but significantly higher PD numbers mark the interfaces between initials and their respective progenies (Zhu et al. 1998a, b). Thus, the multi-initial zone proper exhibits highly abundant PD at its borders and resembles the interface of a single apical cell in this respect. However, such a clear border was not determined in other tracheophyte meristems with IPDs (Imaichi and Hiratsuka 2007; Imaichi et al. 2018) or in the sporophyte meristem of *A. agrestis* (Fig. 5c).

In differentiating tissues of both, *A. agrestis* sporophytes (Fig. 5d) and *A. thaliana* roots (Zhu et al. 1998a, b), PD densities mediating longitudinal transport within cell files were higher than those enabling tangential or radial exchange between cell files. This characteristic pattern of PD densities might establish preferential pathways for positional signaling. Remarkably, fern roots of *A. pinnata*, which grow from merophytes produced by a single initial, also develop the typical PD density pattern without apparent insertion of additional secPD (Gunning 1978).

With the establishment of bryophyte model organisms, enormous progress has been made in unraveling the hormonal and genetic control of meristems in these taxa (Suzuki et al. 2021; Fouracre and Harrison 2022; Frangedakis et al. 2023; Streubel et al. 2023; Flores-Sandoval et al. 2024). Many of the regulators are already known from angiosperms, where they are PD-mobile and act non-cell autonomously, e.g., hormones like auxin (Paterlini 2020; Linh and Scarpella 2022), members of several transcription factor families (WOX genes, Yadav et al. 2011; KNOX/BELL homeobox genes, Kim et al. 2003; GRAS family, Furuta et al. 2012; LEAFY, Sessions et al. 2000), and small RNA species (Furuta et al. 2012). Thus, knowledge of PD network diversity among gametophytic and sporophytic embryophyte meristems (Fig. S1) is indispensable for our conception of the evolutionary development of meristem control.

The present study revealed a typical IPD in the sporophytic hornwort meristem. It clearly differs from the monoplex gametophytic apices of setaphytes with LPDs, which might trigger future research. The similarity of PD networks in sporophyte meristems of hornworts and distinct tracheophyte taxa suggests a homologous origin (Ligrone et al. 2012; Tomescu et al. 2014; Fouracre and Harrison 2022; Fig. S1), but convergent evolution mirroring a common regulatory control of multi-initial (and indeterminately growing) meristems appears also likely. Further investigations, including setaphyte sporophytes, are required for deeper insights. Presumably, PD networks reflect distinct functional demands and signaling pathways of the different meristem types and, thus, might serve as another character trait to make inferences about ancestral structure and functioning of land-plant meristems. The gametophyte-dominant bryophytes might be of particular interest, since they hold a special phylogenetic position as sister to the sporophyte-dominant tracheophytes (Ligrone et al. 2012; Tomescu et al. 2014; Frangedakis et al. 2021; Fouracre and Harrison 2022).

### Supplementary Information

Below is the link to the electronic supplementary material.

Supplementary file1 (DOCX 3693 KB)

Supplementary file2 (MP4 24905 KB)

Supplementary file3 (MP4 39563 KB)

## Data Availability

The data analyzed during this study are included in this manuscript and its supplementary files.

## Abbreviations

FRAP: Fluorescence redistribution (recovery) after photobleaching
IPD: Interface-specific plasmodesmata network
LPD: Lineage-specific plasmodesmata network
PD: Plasmodesmata
secPD: Secondary plasmodesmata
TEM: Transmission electron microscopy
