# Supplementary material for: Plasmodesmata dynamics in bryophyte model organisms: secondary formation and developmental modifications of structure and function
Source: Planta. 2024 Jul 4;260(2):45. doi: 10.1007/s00425-024-04476-1 (PMC11224097; doi:10.1007/s00425-024-04476-1)
Supplement: Supplementary file 1 — Supplementary file1 (DOCX 3693 KB) [file 425_2024_4476_MOESM1_ESM.docx]

PLANTA

Supplementary Information for:

Plasmodesmata dynamics in bryophyte model organisms: secondary formation and developmental modifications of structure and function

Linus Wegner, Katrin Ehlers

Institute of Botany, Justus-Liebig University, D-35392 Giessen, Germany

Correspondence: Linus.Wegner@bot1.bio.uni-giessen.de (LW), Katrin.Ehlers@bot1.bio.uni-giessen.de (KE)

**Figure S1:** Schematic diagram summarizing former hypotheses how evolutionary gains or losses of the ability to form secondary plasmodesmata (secPD) might have influenced the development of different meristem types in land-plant taxa (based on Imaichi and Hiratsuka, 2007; Mansouri, 2012; Evkaikina et al., 2014, 2017; Imaichi et al., 2018; Romanova et al., 2023; adapted to recent phylogenies: Leebens-Mack et al., 2019). The inserted table compares mean PD densities of the meristematic zone and the highest PD densities observed in individual walls (ρ, PD/µm²) between IPDs (blue) and LPDs (red) of tracheophyte shoot and root apical meristems (SAM; Imaichi and Hiratsuka, 2007; RAM; Imaichi et al., 2018), as well as monoplex apical meristems of liverworts and mosses (AM; Mansouri, 2012).


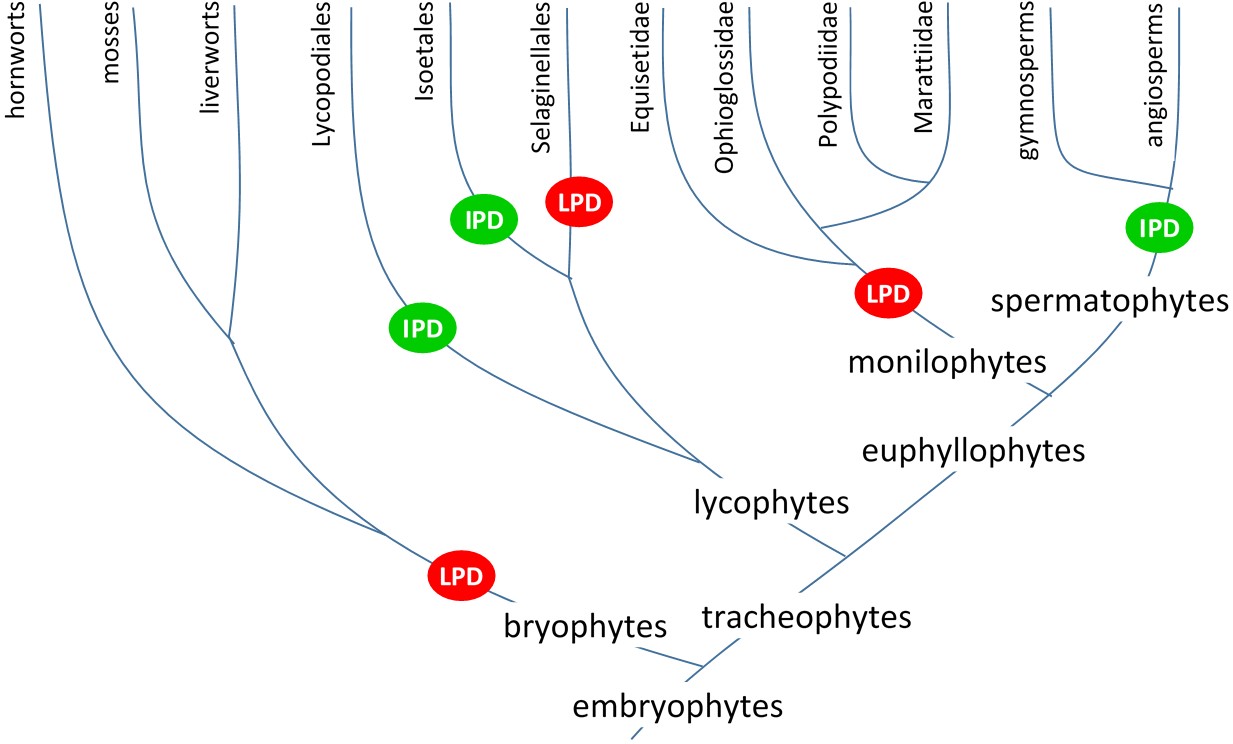


gametophytes sporophytes

**M M M S S M**^†^ **M M M**^†^ **M**^†^ **S**^‡^ **D**


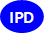

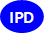

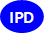


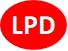

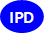
Throughout the land-plant taxa, simplex (S) and duplex meristems (D) with multiple initials are characterized by interface-specific PD networks (        ) in which low PD densities remain constant during development. This is most likely due to secPD formation. Monoplex meristems with a single apical cell presumably lack secPD formation and, consequently, exhibit lineage-specific PD networks (        ) with initially high, but declining PD densities. Supposed that the general ability to form secPD varies between the land-plant taxa, evolutionary gains and losses of this trait might have determined the development of distinct types of land-plant meristems according to the following scenarios:


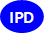
Scenario 1: Most recent common ancestor (MRCA) of tracheophytes lacked secPD formation and the trait developed three times independently in Lycopodiales, Isoetales, and spermatophytes .

Scenario 2: MRCA of tracheophytes lacked secPD formation. The trait evolved independently in lycophytes and spermatophytes , and was subsequently lost in Selaginellales .

Scenario 3 (and 4): MRCA of tracheophytes (or even embryophytes) featured secPD formation      . The trait was subsequently lost in Selaginellales and monilophytes (and eventually bryophytes) .

Our present findings (yellow arrow) suggest that bryophyte gametophytes are able to form secPD in maturating tissues and this trait was most likely present in the MRCA of bryophytes (or even embryophytes). We raise the alternative hypothesis that land-plant taxa with monoplex meristems and LPDs do not necessarily lack the general capacity to form PD secondarily. SecPD formation may purposely not take place in these meristems to guarantee a particularly high symplasmic connectivity of the apical cell and its direct neighbours, which might be required for the maintenance of the initial and its indeterminate growth.

^†^ Occurrence of a few initials was sometimes reported for (older) apices of Marattiales (Marattiidae) and Osmundales (Polypodiidae) (Romanova et al., 2023) as well as for Selaginellales (Harrison et al., 2007; Fouracre and Harrison, 2022). However, other authors observed monoplex apices in these taxa (White and Turner, 1995; Jones and Drinnan, 2009; Imaichi et al., 2018).

^‡^ Occurrence of duplex meristems was reported for Gnetopsida (gymnosperms) (Romanova et al., 2023)


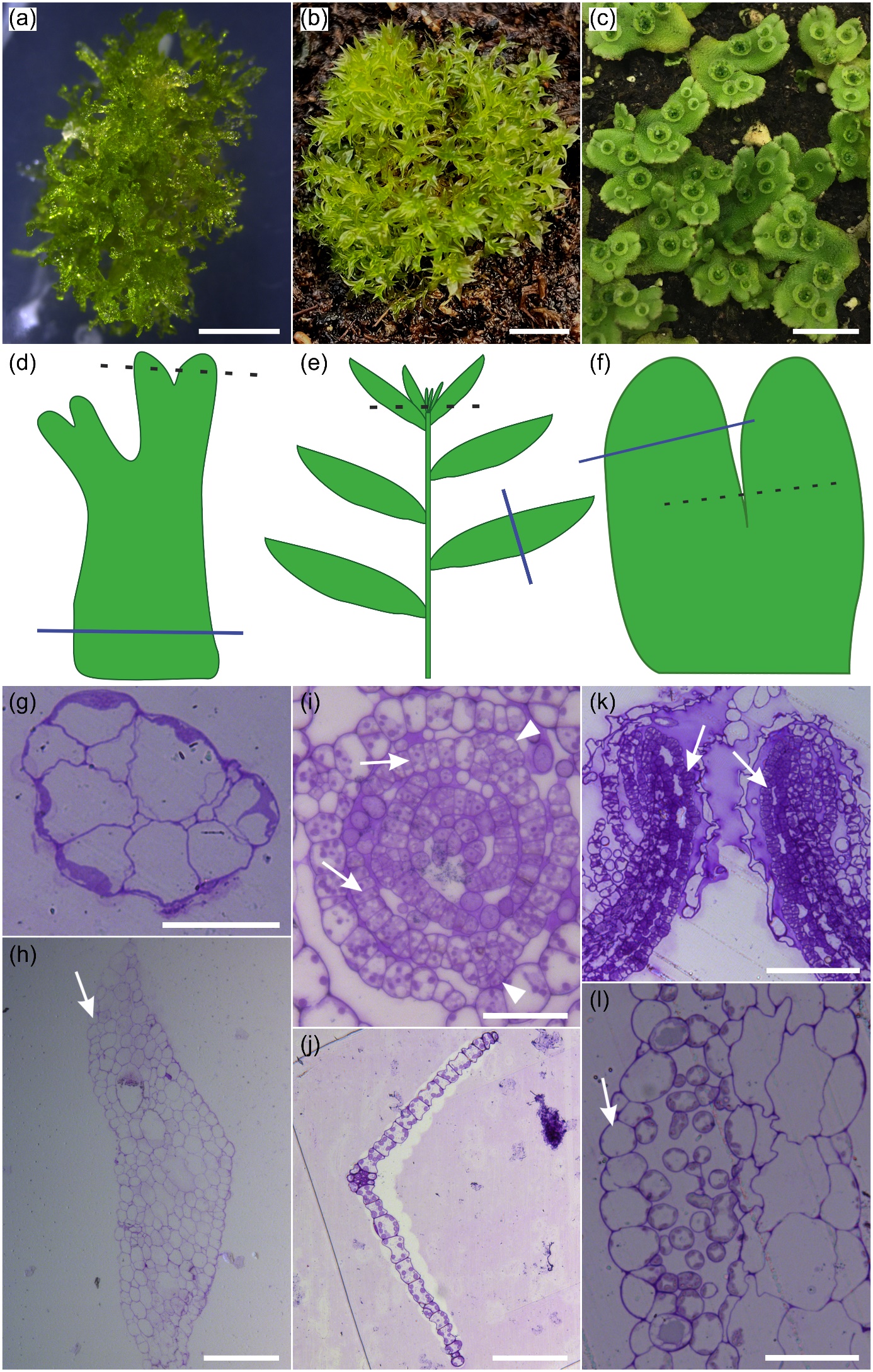
**Figure S2:** Bryophyte model organisms and sample selection for PD counts. Images of (**a**) *Anthoceros agrestis* (hornwort) thalloid gametophytes, (**b**) *Physcomitrium patens* (moss) gametophores with phyllids and (**c**) *Marchantia polymorpha* (liverwort) thalloid gametophytes (with gemmae cups for asexual reproduction). (**d**-**f**) Corresponding schematic drawings marking the regions which were selected for young (black dashed line) and mature (blue solid line) tissue samples. (**g**-**l**) The exact positions of the TEM sections were chosen with the help of light-microscopic analyses rather than merely based on the distance from the apical meristem. For *A. agrestis*, the young tissues (g), which were cross-sectioned directly underneath the thallus tip, were composed of only two vacuolated cell layers with only miniscule intercellular spaces. Yet, the mature thallus regions (h), sectioned several mm underneath the tip, were composed of at least six homogenous parenchyma layers with very large intercellular spaces, covered by upper (arrow) and lower epidermal layers. For *P. patens*, the gametophore tips were cross-sectioned (i) and median regions of the inner developing phyllids (arrows), whose cells were already partially vacuolated, and which show an identifiable developing hadrom (arrowheads), were chosen as young tissue samples. Central regions of mature phyllids (j) protruding orthogonally from the median region of the cauloid were also selected for TEM analysis. For *M. polymorpha*, the young cross-sectioned thallus tissues chosen for analyses (k, arrows) were separated from the apical cell in the meristematic notch by at least ten cell layers. Cells were already partially vacuolated, and had started to form the intercellular spaces, which later develop into the air chambers. The complex mature tissues (l, arrow), cut from the median thallus region between the meristematic notch and the tips of the bifurcated thallus, were characterized by fully developed air chambers and a clear distinction between the upper assimilatory tissue and the storage parenchyma at the bottom, which was composed of at least two cell layers. Plasmodesmata were counted in anticlinal walls of upper epidermal cells (arrows in h,k,l,), excluding non-epidermal cells, like hadrom cells in *P. patens* phyllids and air pores in *M. polymorpha*. Scale bars in (a): 2 mm, (b): 5 mm, (c): 1 cm (g,h,l): 50 µm, (i,k): 100 µm, (j): 200 µm


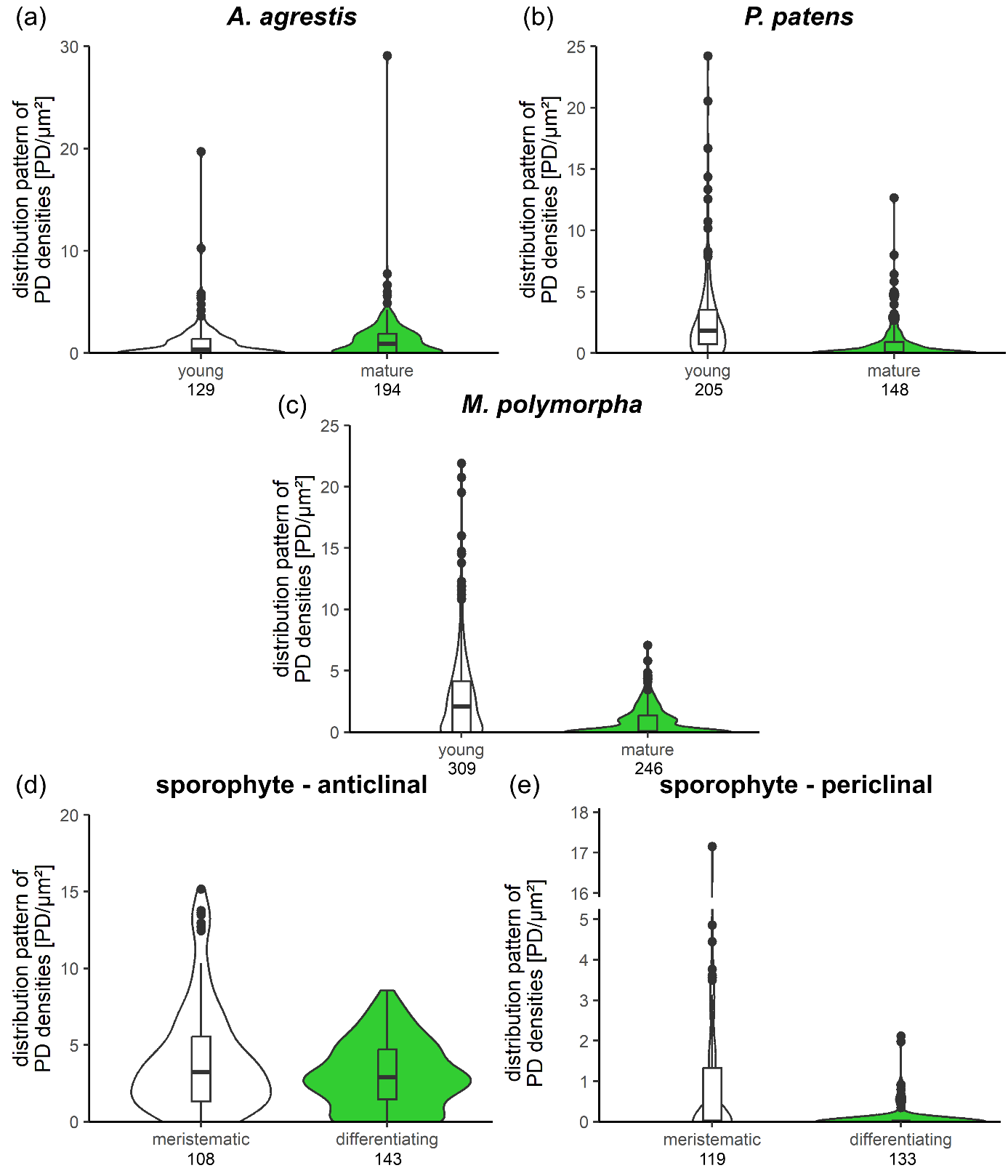


**Figure S3:** Distribution patterns of PD densities in individual walls were analyzed in different bryophyte species and in distinct developmental stages. (**a**) Distribution patterns were alike in young and mature anticlinal epidermal walls of thalloid *A. agrestis* gametophytes. (**b**) In anticlinal walls of young *P. patens* phyllids, the majority of walls showed an even distribution of PD densities between ~0.5 and 4 PD/µm², but increased numbers of walls with lower PD densities occurred in mature phyllids. (**c**) Similar developmental changes were also observed with the distribution patterns in anticlinal epidermal walls of thalloid *M. polymorpha* gametophytes. The majority of young walls possessed between ~0 and 4.5 PD/µm², whereas the walls of mature thalli are characterized by lower PD densities or complete lack of PD in the observed sections. (**d**) For *A. agrestis* sporophytes, the distribution patterns of PD densities in individual walls showed only minor differences between anticlinal walls of meristematic and differentiating cells. (**e**) In periclinal walls of meristematic cells, the PD densities were already less evenly distributed and generally reduced, but the abundance of walls without any PD in the observed sections was strongly increased in differentiating cells. The maximum density observed in an individual wall of the sporophyte meristem was 17.154 PD/µm².

In each graph, the PD densities of individual walls from three biological replicates were merged and the total number of observed walls is shown below.


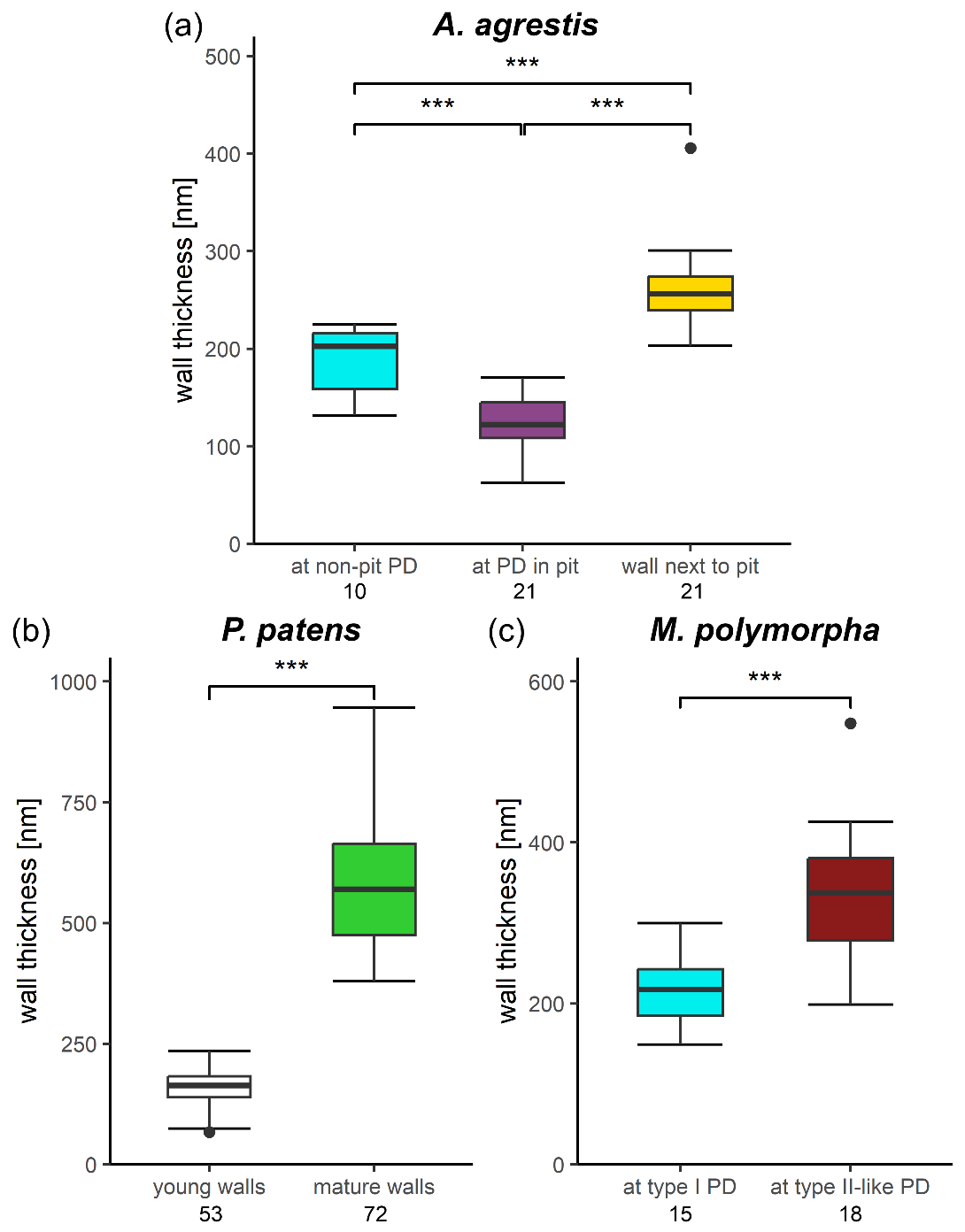


**Figure S4:** Correlation between wall thickness and PD structure in *A. agrestis*, *P. patens,* and *M. polymorpha*. (**a**) At sites, where PD traversed non-pitted regions of the anticlinal walls between mature *A. agrestis* epidermal cells, the walls were only 188.4±33.6 nm (mean±standard deviation) thick, so that pit formation was probably not required (cyan). In pitted wall regions, PD (violet) were located in thinner ‘pit membranes’ (121.4±28.3 nm), but the wall regions surrounding the pit chambers (yellow) were significantly thicker (258.7±42.5 nm). (**b**) The thin walls (159.1±39.4 nm) between young cells of *P. patens* phyllids (white) contained almost exclusively type I PD, while, at maturity (green), cell walls were much thicker (578.8±124.6 nm) and 65% of the PD showed a type II-like morphology with visible cytosolic sleeves (see Figure 2l). (**c**) Similarly, anticlinal wall regions of mature *M. polymorpha* epidermal cells, which were traversed by type I PD, were relatively thin (216.1±47.4 nm, cyan), and in wall areas which were thicker than 300 nm, only type II-like PD were identified (341.6±81.5 nm, red). Significance levels (*** p<0.001) were calculated with a Kruskal-Wallis test followed by pairwise Wilcoxon rank-sum tests for *A. agrestis*, a Wilcoxon rank-sum test for *P. patens*, and a two-sample Student’s t-test for *M. polymorpha*. The total numbers of measured wall sites are depicted below the respective graphs.


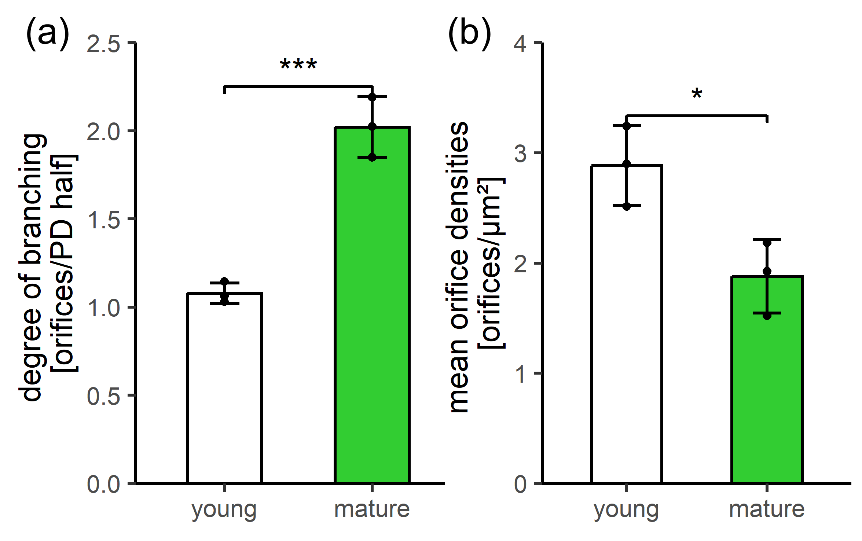


**Figure S5:** Quantitative data on PD branching obtained for anticlinal *M. polymorpha* epidermal walls in the young and mature developmental stage. (**a**) The degree of PD branching (orifices/PD half; Table S3) increased to almost double values, due to the formation of (complex) branched PD in the course of development. (**b**) Despite the increase in the degree of PD branching (a) and the resulting increase in orifice frequencies (Figure 4c), orifice densities (orifices/µm²; Table S3) still decreased during wall expansion, but not to the same extent as PD densities (Figure 4a).

Graphs show means, standard deviations, and data points of three biological replicates each, with a total of 309 and 246 cell walls analyzed, respectively. Significance levels (* p<0.05, *** p<0.001) were calculated with two-sample Student’s t-tests.


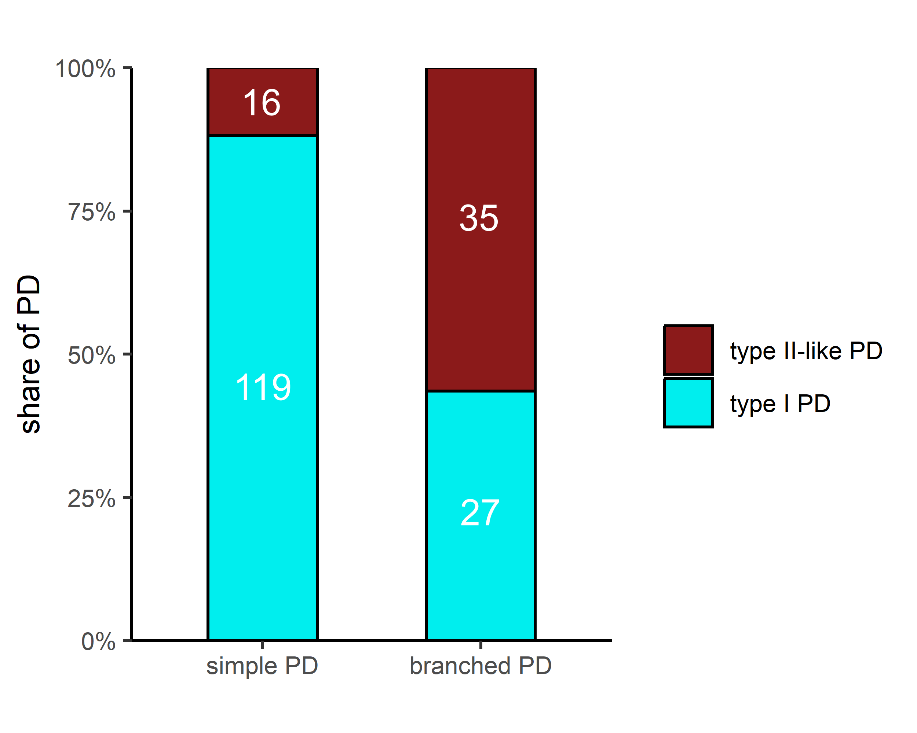


**Figure S6:** Correlation between the characters ‘PD branching’ and ‘type I-to-type II transition’ in mature anticlinal epidermal walls of *M. polymorpha*. Percentages of the total numbers of simple or branched PD, which exhibit a type I or type II-like morphology, observed with all PD in a merged data set of the three replicates. The majority of the simple unbranched PD (86.23%) had a type I morphology, while more than half of the (complex) branched PD possessed a type II-like morphology (56.45%). Numbers of analyzed PD are shown in the bar charts.


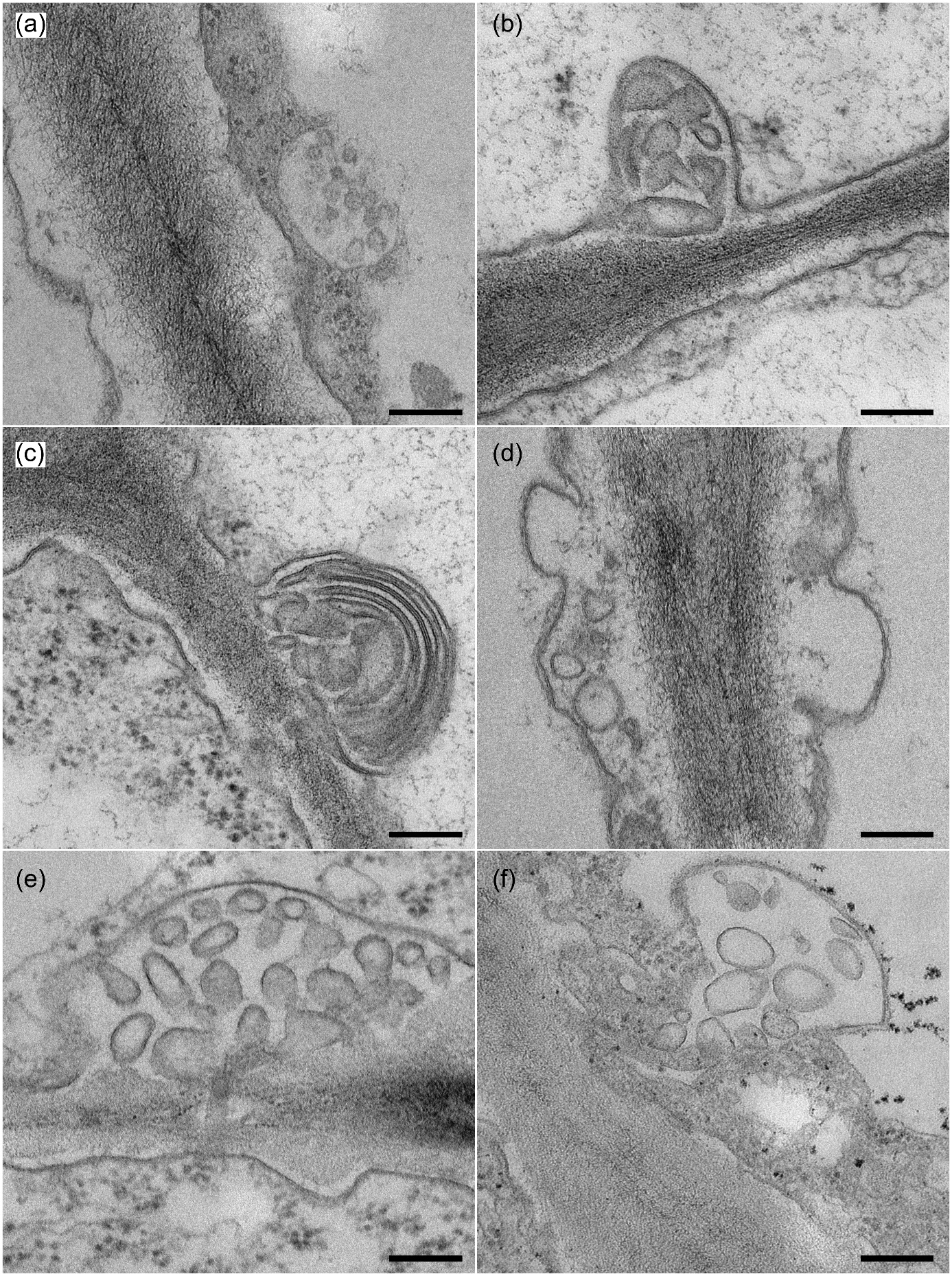


**Figure S7:** High abundance of vesicles and multivesicular bodies (MVB) at bryophyte walls. In TEM sections, vesicles and MVB were frequently observed in (**a-d**) *A. agrestis*, (**e**) *P. patens*, and (**f**) *M. polymorpha.* Vesicles and MVB were found to approach the cell wall (a,f) or fuse with it (b-e), sometimes in close vicinity to PD (c,e). The frequency of these observations was much higher than in seed plants and was detected in all bryophytes to similar extents. This suggests that, in parallel to symplasmic exchange via PD, transport via (extracellular) vesicles may possibly play an important role in the non-seed plants and it might be interesting to trace this hypothesis in future studies. Scale bars: 200 nm

**Table S1:** Overview of published reports related to secPD formation, PD proteome, genome (PD associated proteins), and PD transport capabilities of the three major bryophyte taxa. If not mentioned otherwise, reports refer to gametophytic tissues.

|  | hornworts | liverworts | mosses |
| --- | --- | --- | --- |
| secPD formation | - abundant PD in derivatives of the apical initial in monoplex meristem (but abundant PD in initial wall can also be seen in Fig. 8A) - *Leiosporoceros dussii* (Villarreal Aguilar, 2006) - abundant PD in basal meristem and decreasing abundance with cell differentiation (but no PD counts or TEM images shown) - *Phaeomegaceros fimbriatus* basal meristem **sporophyte** (Villarreal Aguilar, 2006) | - LPD w/o secPD formation in monoplex meristem - *Haplomitrium gibbsiae*, *Pellia epiphylla*, *Metzgeria conjugate*, *Bazzania triolobata* (Mansouri, 2012) | - LPD w/o secPD formation in monoplex meristem - *Takakia ceratophylla, Takakia lepidozioides* (Mansouri, 2012) - LPD w/o secPD formation in apical and subapical meristems - *Sphagnum cuspidatum* (Ligrone and Duckett, 1998) |
|  |  | - branched PD (interpreted by others as indication for secPD formation) - *Monoclea gottschei* thallus (Cook et al., 1997) - branched PD *Asterella wilmsii* food conducting cells shown in Fig. 5G,H (Ligrone and Duckett, 1994a) - branched PD *Conocephalum conicum* thallus in Figure 33 (Ligrone et al., 2008) - Water-conducting cells, initial elongation with sharp decrease of PD densities followed by an increase in orifice densities due to formation of branched PD - *Symphyogyna brasiliensis* (Ligrone and Duckett, 1996) → authors state: no *de-novo* PD formation, but branching as modification of existing PD - complex branched PD - *Monoclea forsteri* **sporophyte** foot cells (Carafa et al., 2003) | - secPD formation keeps PD density constant - developing *Sphagnum palustre* phyllids (Schnepf and Sych, 1983) |
| PD proteome |  | - multiple GHL17s - *Marchantia polymorpha* cell wall proteome (Kolkas et al., 2022) | - MCTPs, GHL17s, exordium-like proteins, tetraspanins, synaptotagmin - *Physcomitrium patens* protonema (Gombos et al., 2023)  **no** CALSs, PDLPs, PDCBs - 1 MCTP, 3 GHL17s (PDBGs), 1 tetraspanin - *Physcomitrium patens* mix of gametophore and protonema (Johnston et al., 2023)  **no** PDLPs and CALSs |
| genome  (PD associated proteins) | - *Anthoceros agrestis* genome (Li et al., 2020) | - **no** PDLPs - *Marchantia polymorpha* genome (Vaattovaara et al., 2019) - 2 CALSs, 26 β-1,3-glucanases, 2 PDBGs, 2 TETs (no PD association shown) - *Marchantia polymorpha* genome (Table S10 in Bowman et al., 2017)  **no** PDLPs, PDCBs, BG_ppap | - **no** PDLPs - *Physcomitrium patens* genome (Vaattovaara et al., 2019) - GHL17s - *Physcomitrium patens* genome (Gaudioso-Pedraza and Benitez-Alfonso, 2014) - Genome *Physcomitrium patens* PEATmoss (Fernandez-Pozo et al., 2020) - e.g. PDCB5 (Pp3c19_12230V3.1), MCTP2 (twice; Pp3c16_9260V3.1, Pp3c27_540V3.1), CALS4 (Pp3c20_20530V3.1), CALS5 (Pp3c10_19330V3.1), CALS9 (twice; Pp3c10_20867V3.1, Pp3c10_16403V3.1), CALS10 (Pp3c10_16400V3.1)  **no** PDLPs - near telomere-to-telomere *Physcomitrium patens* genome (Bi et al., 2024) |
| transport though PD |  |  | - dendra2 (26.1 kDa) very slow transport in **phyllids**, but free transport in **protonema** (disrupted by ABA application, increased by sodium azide) - *Physcomitrium patens* (Kitagawa and Fujita, 2013, 2015; Kitagawa et al., 2019) - CF(DA) (376 Da) transport in protonema - *Physcomitrium patens* (Gombos et al., 2023) |

Frequent observations of paired PD in multiple studies (e.g. Schnepf, 1973; Ligrone and Duckett, 1994b; Ligrone et al., 2000; Mansouri, 2012) might be interpreted as indications for secPD formation by PD twinning. However, studies predated the discovery of the twinning mechanism.

**Table S2:** Previous morphological descriptions of developing and mature PD in bryophytes observed by TEM.

| literature | species | tissue | PD modification |  |
| --- | --- | --- | --- | --- |
| Liverworts | | | | |
| (Mansouri, 2012) | *Haplomittrium gibbsiae*  (Haplomitriopsida, Calobryales)  *Pellia epiphylla*  (Jungermanniopsida, Pelliales)  *Metzgeria conjugata*  (Jungermanniopsida, Metzgeriales)  *Bazzania trilobata*  (Jungermanniopsida, Jungermanniales) | apical cell and derivatives | no peculiarities |  |
| (Cook et al., 1997) | *Monoclea gottschei*  (Marchantiopsida, Marchantiales) | thallus | **branched** PD (X- and Y-shaped) without median dilatations |  |
| (Ligrone et al., 2008) | *Conocephalum conicum*  (Marchantiopsida, Marchantiales) | thallus | **PD in** **pits**  **branched** PD (Y-shaped) with **median dilatations** and bloated DT (Figure 33) |  |
| (Ligrone and Duckett, 1994a) | *Asterella wilmsii*  (Marchantiopsida,Marchantiales) | food-conducting cells | **PD in** **pits**  **branched** PD (Y-shaped) with **median dilatations** and bloated DT (Figure 5G,H) ^†^ |  |
| (Ligrone et al., 2000) | *Asterella wilmsii*  (Marchantiopsida,Marchantiales) | food-conducting cells | **PD in pits**  PD with **median dilatations** |  |
| (Renzaglia et al., 2007) | *Haplomitrium blumei*  *Haplomitrium gibbsae*  (Haplomitriopsida, Calobryales) | food-conducting cells | slightly enlarged PD (no image shown) |  |
| (Ligrone and Duckett, 1996) | *Symphyogyna brasiliensis*  (Jungermanniopsida, Pallavinciniales) | water conducting cells | **branched** PD → in mature walls open pores (called ’pits’) |  |
| (Ligrone et al., 2002) | *Symphyogyna undulata*  *Hymenophyton flabellatum*  (Jungermanniopsida, Pallavinciniales) | water conducting cells | PD-derived pores (partially still responsive to callose label) |  |
| (Ligrone et al., 2000) | *Haplomitrium hookeri*  (Haplomitriopsida, Calobryales)  *Symphyogyna*  *Hymenophyton*  *Pallavicinia*  (Jungermanniopsida, Pallavinciniales) | water-conducting cells | PD-derived pores |  |
| (Carafa et al., 2003) | *Monoclea forsteri*  (Marchantiopsida,Marchantiales) | sporophyte foot cells | **complex branched** PD with **median dilatations** and bloated DT |  |
| Mosses | | | | |
| (Mansouri, 2012) | *Takakia ceratophylla*  *Takakia lepidozoides*  (Takakiopsida, Takakiales) | apical cell and derivatives | no peculiarities |  |
| (Schnepf, 1973)  (Schnepf and Sych, 1983) | *Sphagnum palustre*  (Sphagnopsida, Sphagnales) | developing leaflets | no peculiarities (Figures 11-14,16,20,21) |  |
| (Ligrone and Duckett, 1998) | *Sphagnum cuspidatum*  *Sphagnum magellanicum*  and other *Sphagnum* species  (Sphagnopsida, Sphagnales) | “subapical meristem of leafy stem” | PD located **in pits** only during wall elongation  **median dilatations** with bloated DT (Figures 3e,f, 5f) |  |
| (Stevenson, 1974) | *Atrichum undulatum*  (Polytrichopsida, Polytrichales) | leptoids and surrounding parenchyma cells | in parenchyma cells: PD with **median dilatations** with bloated DT  leptoids: ‘pores’ with twice the diameter and more strongly pronounced **median dilatation** |  |
| (Pressel et al., 2006) | *Polytrichum formosum*  (Polytrichopsida, Polytrichales) | leptoids and parenchyma cells | **median dilatations** with bloated DT |  |
| (Ligrone et al., 2000) | *Sphagnum recurvum*  (Sphagnopsida, Sphagnales)  *Aulacomnium palustre*  (Bryopsida, Bryales)  *Polytrichum formosum*  (Polytrichopsida, Polytrichales) | leptoids (food-conducting cells) | **median dilatations** with bloated DT |  |
|  | *Sphagnum cuspidatum*  (Sphagnopsida, Sphagnales)  *Mnium hornum*  *Plagiomnium undulatum*  (Bryopsida, Bryales)  *Polytrichum juniperinum*  (Polytrichopsida, Polytrichales) | leptoids (food-conducting cells) | **median dilatations** with bloated DT:   - *M. hornum* only slightly enlarged DT with visible cytosolic sleeve - *S. cuspidatum* not mentioned but visible in Fig. 8d - *P. undulatum* & *P. juniperinum* insufficient magnification of Figure 7a |  |
| (Ligrone and Duckett, 1994b) | *Mnium hornum*  *Plagiomnium undulatum*  (Bryopsida, Bryales) | leptoids (food-conducting cells) | **median dilatations** (partially bloated DT) |  |
|  | *Atrichum undulatum*  (Polytrichopsida, Polytrichales) | leptoids (food-conducting cells) | no dilatation mentioned, but Figure 6I depicts slight dilatation with bloated DT |  |
| (Scheirer, 1978) | *Dendroligotrichum dendroides*  (Polytrichopsida, Polytrichales) | leptoids (food-conducting cells) | PD with **median dilatations** and constricted DT |  |
| (Ligrone and Duckett, 1994b) | *Polytrichum formosum*  (Polytrichopsida, Polytrichales) | leptoids (food-conducting cells) | slight **median dilatations** (multiple membranous tubules pass though PD) |  |
| (Renzaglia et al., 2007) | *Takakia ceratophylla*  (Takakiopsida, Takakiales) | water-conducting cells | PD-derived pores |  |
| (Ligrone et al., 2002) | *Takakia lepidoziodes*  *Takakia ceratophylla*  (Takakiopsida, Takakiales) | water conducting cells | PD-derived pores (partially still responsive to callose label) |  |
| (Ligrone et al., 2000) | *Polytrichum formosum*  (Polytrichopsida, Polytrichales) | water-conducting cells | PD-derived pores |  |
| (Cook et al., 1997) | *Sphagnum fimbriatum*  (Sphagnopsida, Sphagnales) | protonema | no peculiarities (spokes visible in cross-sections) |  |
| (Kitagawa et al., 2019) | *Physcomitrium patens*  (Bryopsida, Funariales) | protonema | no peculiarities |  |
| (Gombos et al., 2023) | *Physcomitrium patens*  (Bryopsida, Funariales) | protonema | no peculiarities |  |
| (Schnepf and Sawidis, 1991) | *Funaria hygrometrica*  (Bryopsida Funariales) | chloronema fragmented by tmema cells | sometimes small **median dilatations** with constricted DT (Fig. 2); PD later occluded |  |
| (Gambardella and Alfano, 1990) | *Timmiella barbuloides*  (Bryopsida, Pottiales) | dividing archesporium (sporophyte) | no peculiarities (Figures 1,5,6,17) |  |
| Hornworts | | | | |
| (Villarreal Aguilar, 2006) | *Leiosporoceros dussii*  (Leiosporocerotopsida, Leiosporocerotales) | gametophyte meristem | no peculiarities (Figure 8A) |  |
| (Cook et al., 1997) | *Notothylas orbicularis*  (Anthocerotopsida, Notothyladales) | gametophyte | no peculiarities (spokes visible in cross-sections) |  |
| (Villarreal Aguilar and Renzaglia, 2006)  (Villarreal Aguilar, 2006) | *Phaeomegaceros fimbriatus*  (Anthocerotopsida, Dendrocerotales) | gametophyte cell close to placenta  foot parenchyma cells (sporophyte)  sporophyte basal meristem | no peculiarities (Figures 13C, 14F, 16D and 3C, 4F, 6D) |  |
| (Ligrone and Renzaglia, 1990) | *Dendroceros tubercularis*  (Anthocerotopsida, Dendrocerotales) | foot parenchyma cells (sporophyte) | slightly dilated PD (Figure 2b) |  |

^†^ quotation: ‘[these PD] with their highly expanded cytoplasmic annuli and clear associations with ER, bear a most striking resemblance to some of the most highly differentiated plasmodesmatal configurations found in vascular plants.’

**Table S3:** Data obtained from the PD counts on bryophyte gametophytes performed by TEM. For all bryophyte species and each developmental stage, the collected data are shown per biological replicate. Depending on the parameter, the sums (for quantities) or mean values (for derived parameters) of the replicates were calculated (Σ / mean with standard deviation). This includes the total numbers of analyzed walls (nWalls), observed PD (nPD), and PD orifices (nOriW). The numbers (n) of branched, twinned, and type II-like PD as well as their shares (%) out of the total number of PD are depicted. The total wall length of the analyzed walls (µm) was used to calculate PD densities (ρ(PD); PD/µm² cell wall) and orifice densities (ρ(ori); orifices/µm² cell wall), as well as mean cell-interface areas (µm²). These derived parameters were used to calculate PD frequencies (f(PD); total number of PD/average interface area) and orifice frequencies (f(ori); total number of orifices/average interface area). The degree of branching (orifices/PD half) was used to calculate ρ(ori) and f(ori).

|  | nWalls | nPD | nOriW | branched PD | | twinned PD | | type II-like PD | | total wall  length | ρ(PD) | ρ(ori) | mean interface area | f(PD) | f(ori) | degree of branching |
| --- | --- | --- | --- | --- | --- | --- | --- | --- | --- | --- | --- | --- | --- | --- | --- | --- |
|  |  |  |  | n | % | n | % | n | % |  |  |  |  |  |  |  |
| ***A. agrestis***  **young**  gametophyte  upper epidermis | 29 | 53 | 93 | 7 | 13.21% | 17 | 32.08% | 0 | 0.00% | 367.872 | 1.315 | 1.772 | 508.07 | 667.90 | 900.21 | 1.35 |
|  | 58 | 97 | 144 | 4 | 4.12% | 33 | 34.02% | 0 | 0.00% | 921.997 | 0.986 | 1.118 | 636.69 | 627.56 | 711.57 | 1.13 |
|  | 42 | 79 | 97 | 2 | 2.53% | 7 | 8.86% | 0 | 0.00% | 523.665 | 1.421 | 1.515 | 499.38 | 709.64 | 756.43 | 1.07 |
| Σ / mean | 129 | 229 | 334 | 13 | 6.62% | 57 | 24.99% | 0 | 0.00% | 1813.534 | 1.240 | 1.468 | 548.04 | 668.37 | 789.40 | 1.18 |
| standard deviation |  |  |  |  | ±5.76 |  | ±14.00 |  | - |  | ±0.227 | ±0.330 | ±76.89 | ±41.04 | ±98.55 | ±0.15 |
| ***A. agrestis***  **mature**  gametophyte  upper epidermis | 30 | 41 | 54 | 1 | 2.44% | 4 | 9.76% | 0 | 0.00% | 648.775 | 0.582 | 0.684 | 3177.64 | 1850.59 | 2172.43 | 1.17 |
|  | 108 | 252 | 455 | 30 | 11.90% | 65 | 25.79% | 0 | 0.00% | 1473.684 | 1.525 | 2.035 | 2004.99 | 3057.35 | 4079.46 | 1.33 |
|  | 22 | 90 | 139 | 13 | 14.44% | 2 | 2.22% | 0 | 0.00% | 351.437 | 2.337 | 3.349 | 2347.23 | 5485.17 | 7860.19 | 1.43 |
|  | 34 | 52 | 96 | 5 | 9.62% | 13 | 25.00% | 0 | 0.00% | 880.072 | 0.530 | 0.678 | 3803.39 | 2015.87 | 2580.31 | 1.28 |
| Σ / mean | 194 | 435 | 744 | 49 | 9.60% | 84 | 15.69% | 0 | 0.00% | 3353.968 | 1.244 | 1.686 | 2833.31 | 3102.24 | 4173.10 | 1.31 |
| standard deviation |  |  |  |  | ±5.16 |  | ±11.62 |  | - |  | ±0.860 | ±1.279 | ±812.81 | ±1676.03 | ±2591.21 | ±0.11 |
| ***P. patens***  **young**  gametophyte  phyllids | 78 | 250 | 381 | 2 | 0.80% | 48 | 19.20% | 0 | 0.00% | 758.599 | 2.961 | 3.008 | 136.77 | 404.92 | 411.40 | 1.02 |
|  | 67 | 134 | 219 | 5 | 3.73% | 45 | 33.58% | 4 | 2.99% | 600.118 | 2.013 | 2.140 | 154.11 | 253.61 | 269.62 | 1.06 |
|  | 60 | 183 | 297 | 2 | 1.09% | 35 | 19.13% | 0 | 0.00% | 657.501 | 2.566 | 2.619 | 125.96 | 395.42 | 403.57 | 1.02 |
| Σ / mean | 205 | 567 | 897 | 9 | 1.87% | 128 | 23.97% | 4 | 1.00% | 2016.218 | 2.513 | 2.589 | 138.95 | 351.32 | 361.53 | 1.03 |
| standard deviation |  |  |  |  | ±1.61 |  | ±8.32 |  | ±1.73 |  | ±0.476 | ±0.434 | ±14.20 | ±84.75 | ±79.69 | ±0.03 |
| ***P. patens***  **mature**  gametophyte  phyllids | 40 | 45 | 68 | 1 | 2.22% | 5 | 11.11% | 30 | 66.67% | 452.65 | 0.708 | 0.802 | 913.04 | 646.14 | 732.29 | 1.13 |
|  | 65 | 66 | 79 | 0 | 0.00% | 6 | 9.09% | 44 | 66.67% | 623.53 | 0.759 | 0.759 | 773.98 | 587.14 | 587.14 | 1.00 |
|  | 43 | 59 | 73 | 0 | 0.00% | 23 | 38.98% | 37 | 62.71% | 500.13 | 0.767 | 0.767 | 938.43 | 719.90 | 719.90 | 1.00 |
| Σ / mean | 148 | 170 | 220 | 1 | 0.74% | 34 | 19.73% | 111 | 65.35% | 1576.31 | 0.744 | 0.776 | 875.15 | 651.06 | 679.78 | 1.04 |
| standard deviation |  |  |  |  | ±1.28 |  | ±16.70 |  | ±2.29 |  | ±0.032 | ±0.023 | ±88.53 | ±66.52 | ±80.46 | ±0.08 |
| ***M. polymorpha***  **young**  gametophyte  upper epidermis | 102 | 214 | 356 | 6 | 2.80% | 41 | 19.16% | 0 | 0.00% | 730.211 | 2.728 | 2.899 | 75.94 | 207.14 | 220.12 | 1.06 |
|  | 108 | 186 | 373 | 12 | 6.45% | 50 | 26.88% | 0 | 0.00% | 632.979 | 2.833 | 3.241 | 62.17 | 176.10 | 201.49 | 1.14 |
|  | 99 | 174 | 303 | 3 | 1.72% | 34 | 19.54% | 0 | 0.00% | 678.629 | 2.438 | 2.513 | 72.71 | 177.29 | 182.72 | 1.03 |
| Σ / mean | 309 | 574 | 1032 | 21 | 3.66% | 125 | 21.86% | 0 | 0.00% | 2041.819 | 2.666 | 2.884 | 70.27 | 186.84 | 201.44 | 1.08 |
| standard deviation |  |  |  |  | ±2.48 |  | ±4.35 |  | - |  | ±0.204 | ±0.364 | ±7.20 | ±17.59 | ±18.70 | ±0.06 |
| ***M. polymorpha***  **mature**  gametophyte  upper epidermis | 57 | 58 | 159 | 17 | 29.31% | 7 | 12.07% | 7 | 12.07% | 417.067 | 1.181 | 2.183 | 234.93 | 277.41 | 512.89 | 1.85 |
|  | 94 | 65 | 164 | 19 | 29.23% | 7 | 10.77% | 18 | 27.69% | 765.875 | 0.752 | 1.523 | 261.60 | 196.75 | 398.37 | 2.02 |
|  | 95 | 74 | 219 | 26 | 35.14% | 6 | 8.11% | 26 | 35.14% | 745.001 | 0.880 | 1.926 | 251.79 | 221.46 | 484.99 | 2.19 |
| Σ / mean | 246 | 197 | 542 | 62 | 31.23% | 20 | 10.32% | 51 | 24.97% | 1927.943 | 0.938 | 1.877 | 249.44 | 231.88 | 465.42 | 2.02 |
| standard deviation |  |  |  |  | ±3.39 |  | ±2.02 |  | ±11.77 |  | ±0.220 | ±0.333 | ±13.49 | ±41.33 | ±59.72 | ±0.17 |

**Table S4:** Data obtained from the PD counts on *A. agrestis* sporophytes performed by TEM. For anticlinal and periclinal walls of meristematic and differentiating cells, the collected data are shown per biological replicate. Depending on the parameter, the sums (for quantities) or mean values (for derived parameters) of the replicates were calculated (Σ / mean with standard deviation). This includes the total numbers of analyzed walls (nWalls), observed PD (nPD), and PD orifices (nOriW). The numbers (n) of branched, twinned, and type II-like PD as well as their shares (%) out of the total number of PD are depicted. The total wall length of the analyzed walls (µm) was used to calculate PD densities (ρ(PD); PD/µm² cell wall) and orifice densities (ρ(ori); orifices/µm² cell wall), as well as mean cell-interface areas (µm²). These derived parameters were used to calculate PD frequencies (f(PD); total number of PD/average interface area) and orifice frequencies (f(ori); total number of orifices/average interface area). The degree of branching (orifices/PD half) was used to calculate ρ(ori) and f(ori).

|  | nWalls | nPD | nOriW | branched PD | | twinned PD | | type II-like PD | | total wall length | ρ(PD) | ρ(ori) | mean interface area | f(PD) | f(ori) | degree of branching |
| --- | --- | --- | --- | --- | --- | --- | --- | --- | --- | --- | --- | --- | --- | --- | --- | --- |
|  |  |  |  | n | % | n | % | n | % |  |  |  |  |  |  |  |
| **anticlinal walls meristematic**  *A. agrestis*  sporophyte | 38 | 198 | 295 | 1 | 0.51% | 34 | 17.17% | 0 | 0.00% | 346.013 | 5.217 | 5.271 | 51.67 | 269.56 | 272.33 | 1.01 |
|  | 32 | 74 | 127 | 0 | 0.00% | 4 | 5.41% | 0 | 0.00% | 222.225 | 2.942 | 2.942 |  | 151.00 | 151.00 | 1.00 |
|  | 38 | 107 | 180 | 0 | 0.00% | 21 | 19.63% | 0 | 0.00% | 280.695 | 3.472 | 3.472 |  | 179.38 | 179.38 | 1.00 |
| Σ / mean | 108 | 379 | 602 | 1 | 0.17% | 59 | 14.07% | 0 | 0.00% | 848.933 | 3.877 | 3.895 |  | 200.31 | 201.24 | 1.00 |
| standard deviation |  |  |  |  | ±0.29 |  | ±7.60 |  | - |  | ±1.191 | ±1.221 |  | ±61.52 | ±63.08 | ±0.01 |
| **anticlinal walls differentiating**  *A. agrestis*  sporophyte | 50 | 212 | 302 | 0 | 0.00% | 25 | 11.79% | 0 | 0.00% | 607.502 | 3.078 | 3.078 | 112.41 | 345.98 | 345.98 | 1.00 |
|  | 43 | 220 | 333 | 1 | 0.45% | 25 | 11.36% | 0 | 0.00% | 542.100 | 3.502 | 3.534 |  | 393.67 | 397.25 | 1.01 |
|  | 50 | 226 | 351 | 0 | 0.00% | 28 | 12.39% | 0 | 0.00% | 680.711 | 2.908 | 2.908 |  | 326.91 | 326.91 | 1.00 |
| Σ / mean | 143 | 658 | 986 | 1 | 0.15% | 78 | 11.85% | 0 | 0.00% | 1830.313 | 3.163 | 3.173 |  | 355.52 | 356.71 | 1.00 |
| standard deviation |  |  |  |  | ±0.26 |  | ±0.52 |  | - |  | ±0.306 | ±0.324 |  | ±34.39 | ±36.38 | ±0.01 |
| **periclinal walls meristematic**  *A. agrestis*  sporophyte | 44 | 12 | 16 | 0 | 0.00% | 1 | 8.33% | 0 | 0.00% | 224.477 | 0.473 | 0.473 | 37.60 | 17.77 | 17.77 | 1.00 |
|  | 39 | 21 | 31 | 0 | 0.00% | 8 | 38.10% | 0 | 0.00% | 234.619 | 0.797 | 0.797 | 44.33 | 35.34 | 35.34 | 1.00 |
|  | 36 | 31 | 47 | 0 | 0.00% | 5 | 16.13% | 0 | 0.00% | 217.233 | 1.335 | 1.335 | 44.47 | 59.36 | 59.36 | 1.00 |
| Σ / mean | 119 | 64 | 94 | 0 | 0.00% | 14 | 20.85% | 0 | 0.00% | 676.329 | 0.868 | 0.868 | 42.13 | 37.49 | 37.49 | 1.00 |
| standard deviation |  |  |  |  | - |  | ±15.43 |  | - |  | ±0.435 | ±0.435 | ±3.93 | ±20.88 | ±20.88 | - |
| **periclinal walls differentiating**  *A. agrestis*  sporophyte | 39 | 9 | 15 | 0 | 0.00% | 1 | 11.11% | 0 | 0.00% | 360.524 | 0.220 | 0.220 | 127.00 | 27.90 | 27.90 | 1.00 |
|  | 52 | 7 | 13 | 0 | 0.00% | 1 | 14.29% | 0 | 0.00% | 604.527 | 0.101 | 0.101 | 159.71 | 16.11 | 16.11 | 1.00 |
|  | 42 | 2 | 3 | 0 | 0.00% | 0 | 0.00% | 0 | 0.00% | 769.730 | 0.024 | 0.024 | 251.78 | 6.13 | 6.13 | 1.00 |
| Σ / mean | 133 | 18 | 31 | 0 | 0.00% | 2 | 8.47% | 0 | 0.00% | 1734.781 | 0.115 | 0.115 | 179.50 | 16.71 | 16.71 | 1.00 |
| standard deviation |  |  |  |  | - |  | ±7.50 |  | - |  | ±0.098 | ±0.098 | ±64.70 | ±10.90 | ±10.90 | - |

The average PD density determined for all meristematic walls was 2.576±0.747 PD/µm².

**Table S5:** Additional data serving as quantitative basis for the computations of PD densities and frequencies. For all species and tissue types in the different developmental stages, the average outer PD radius per biological replicate was measured, which was required to calculate PD densities (Gunning, 1978; Methods S3). The numbers of PD measured to calculate this parameter (nR) is also shown. The average height of the analyzed epidermal cells was calculated per replicate from the total wall length, observed in cross sections under the TEM, and the number of analyzed walls depicted in Tables S3,S4. The average cell (wall) length per species and developmental stage was measured in top view light-microscopic images (or in cross sections of *A. agrestis* sporophytes). Numbers of measured walls are mentioned (nCell length). The mean interface area per replicate, used for the frequency calculations, was computed by multiplication of the mean cell height with the mean cell length. The average of these values is given as mean area (per species and developmental stage). For anticlinal walls of the *A. agrestis* sporophytes, the interface areas were directly measured in cross sections, and the numbers of measurements (nArea) used to calculate the mean wall areas are depicted.

| species /  tissue type | developmental stage | PD radius [nm] | nR | mean cell height [µm] | mean cell length [µm] | nCell length | mean interface area per replicate [µm²] | mean area [µm²] |
| --- | --- | --- | --- | --- | --- | --- | --- | --- |
| *Anthoceros agrestis*  gametophyte  upper epidermis | young | 19.73 | 24 | 12.685 | 40.05 | 61 | 508.1 | 548.0 |
|  |  | 17.82 | 36 | 15.897 |  |  | 636.7 |  |
|  |  | 17.44 | 26 | 12.468 |  |  | 499.4 |  |
|  | mature | 19.01 | 23 | 21.626 | 146.94 | 99 | 3177.6 | 2833.3 |
|  |  | 21.43 | 69 | 13.645 |  |  | 2005.0 |  |
|  |  | 19.73 | 22 | 15.974 |  |  | 2347.2 |  |
|  |  | 20.99 | 24 | 25.884 |  |  | 3803.4 |  |
| *Physcomitrium patens*  gametophyte  phyllids | young | 20.88 | 37 | 9.726 | 14.06 | 76 | 136.8 | 138.9 |
|  |  | 20.60 | 32 | 8.957 |  |  | 126.0 |  |
|  |  | 18.98 | 29 | 10.958 |  |  | 154.1 |  |
|  | mature | 40.32 | 27 | 11.316 | 80.68 | 89 | 913.0 | 875.2 |
|  |  | 39.69 | 41 | 9.593 |  |  | 774.0 |  |
|  |  | 49.19 | 36 | 11.631 |  |  | 938.4 |  |
| *Marchantia polymorpha*  gametophyte  upper epidermis | young | 18.29 | 35 | 7.159 | 10.61 | 164 | 75.9 | 70.3 |
|  |  | 15.82 | 23 | 5.861 |  |  | 62.2 |  |
|  |  | 16.77 | 30 | 6.855 |  |  | 72.7 |  |
|  | mature | 25.18 | 27 | 7.317 | 32.11 | 123 | 234.9 | 249.4 |
|  |  | 21.89 | 37 | 8.148 |  |  | 261.6 |  |
|  |  | 21.96 | 32 | 7.842 |  |  | 251.8 |  |
| *Anthoceros agrestis*  sporophyte  periclinal walls | meristematic | 22.09 | 12 | 5.102 | 7.37 | 23 | 37.6 | 41.9 |
|  |  | 21.52 | 20 | 6.016 |  |  | 44.3 |  |
|  |  | 17.94 | 17 | 6.034 |  |  | 44.5 |  |
|  | differentiating | 22.43 | 11 | 9.244 | 13.74 | 24 | 127.0 | 179.2 |
|  |  | 23.22 | 5 | 11.626 |  |  | 159.7 |  |
|  |  | 17.87 | 5 | 18.327 |  |  | 251.8 |  |
|  |  | PD radius [nm] | nR |  | mean wall area [µm²] | nArea |  | |
| *Anthoceros agrestis*  sporophyte  anticlinal walls | meristematic | 19.79 | 47 |  | 51.67 | 24 |  |  |
|  |  | 22.13 | 50 |  |  |  |  |  |
|  |  | 19.87 | 40 |  |  |  |  |  |
|  | differentiating | 22.25 | 33 |  | 112.41 | 25 |  |  |
|  |  | 23.92 | 26 |  |  |  |  |  |
|  |  | 22.77 | 48 |  |  |  |  |  |

**Table S6:** Fluorescence redistribution after photobleaching (FRAP) experiments with *P. patens* phyllids to determine functional PD capacities. Separated for the developmental stage of the phyllids (mature, intermediate, young) and for the fluorescent dye used in the respective experiments (CFDA, 376 Da in the fluorescent CF form; mCherry 26.7 kDa), the table depicts the number of successful bleaching experiments performed, as well as the number and percentage of experiments, in which a fluorescence redistribution occurred. Fluorescence redistribution was regularly observed within 10-20 min, which indicates that the fluorescent dye could move symplasmically via PD from the neighbouring cells into the bleached cells. For mature phyllids, in which mCherry fluorescence redistribution was not observed, the experiments were stopped after a maximum time of 105 min after bleaching. For the bleaching process, argon laser power was set at 50 to 80% and the number of frames were 7 to 60 (0.648s per frame). The settings tended to influence the duration of the lag phase prior to the onset of fluorescence redistribution (see Fig. 3d, green graph).

| age | dye | successful bleaching | redistribution | redistribution [%] |
| --- | --- | --- | --- | --- |
| mature | CFDA | 14 | 11 | 78.57 |
| mature | mCherry | 13 | 0 | 0 |
| intermediate | mCherry | 9 | 2 | 22.22 |
| young | mCherry | 6 | 5 | 83.33 |

**Movie S1:** Successful FRAP with CFDA (376 Da in the fluorescent CF form, λ_ex_=488 nm, λ_em_=505-530 nm) of individual adult cells in a mature *P. patens* phyllid within an observation period of 11 min (min:s). A single cell (arrow) was bleached with 50% argon laser power and 100% intensity of the 476, 488 and 496 nm laser lines for 15 frames (~10s) and fluorescence redistribution started after a short lag phase. Note the nearby cell (*) which had been bleached 10:30 min earlier (with the same laser settings, but for 60 frames (~40s)). Here, fluorescence redistribution could immediately be observed in the depicted time frame. Scale bar: 58 µm

**Movie S2:** Successful FRAP with mCherry (26.7 kDa, λ_ex_=561 nm, λ_em_=590-630 nm) of a small juvenile cell (arrow) in a young *P. patens* phyllid within an observation period of 14:30 min (min:s). A single cell (arrow) and parts of the adjacent cells were bleached with 80% argon laser power and 100% intensity of the 476, 488, 496, and 561 nm laser line for 11 frames (~7s). The redistribution of fluorescence could be tracked starting from the slightly bleached adjacent cells and continuing towards the fully bleached target cell. Scale bar: 15.2 µm

**Methods S1:** TEM fixation protocols. GA: glutardialdehyde; PFA: paraformaldehyde (freshly prepared); NA-P: sodium phosphate buffer; RT: room temperature

| fixation procedure | *Anthoceros agrestis*  gametophyte | *Anthoceros agrestis*  sporophyte | *Physcomitrium patens*  gametophyte | *Marchantia polymorpha*  gametophyte |
| --- | --- | --- | --- | --- |
| sample | thallus tip region  up to 0.5 cm length | whole sporophytes and long, basal segments  in 2% low gelling agarose type VII  (Sigma-Aldrich, Steinheim, Germany) | whole gametophores in 2% low gelling agarose type VII | thallus tip region 0.5-1 cm length |
| fixation | 2.5% GA in  0.05 M Na-P; pH 7.1,  2 h on ice | 2% GA in  0.05 M Na-P;  pH 7.21,  2h at RT +  2h on ice | 2.5% GA in  0.05 M Na-P;  pH 7.23,  2h at RT +  2h on ice | 2.5% GA,  0.5% PFA in  0.05 M Na-P; pH 7.15,  2h at RT +  2h on ice |
| rinsing | 4x in 0.1 M Na-P; pH 7.2 on ice | | | |
| postfixation | 0.9% OsO_4_ in 0.1 M Na-P at 4°C overnight | | | |
| rinsing | 6x in ddH_2_O | | | |
| *en-bloc* staining | 0.5% aqueous uranyl acetate, 2h on ice | | | |
| dehydration | graded ethanol series (30%, 50%, 70%, 90% on ice; 96% 100% at RT)  100% propylene oxide | | | |
| embedding | gradual infiltration with Spurr´s resin (over 2 days) (Spurr, 1969) | | | |
| polymerization | embedding in gelatine capsules or silicon embedding forms  and polymerization at 68°C for 20h | | | |

**Methods S2:** Representativeness of PD counts (and derived parameters), determined in randomly chosen TEM sections of a biological replicate, demands a random PD distribution in the observed cell walls. In order to validate this prerequisite (Imaichi et al., 2018), we analyzed 33 to 38 cell walls of three randomly chosen serial sections of a mature gametophyte sample of *A. agrestis*. The PD densities calculated for the three sections were normally distributed (Shapiro-Wilk test p=0.83) and, for a mean of 1.529 PD/µm², the standard deviation (0.0728 PD/µm²) and coefficient of variation (4.76%) were at a level of divergence that confirmed the validity. Thus, we adopted the method of Imaichi et al. (2018) for our study, and used representative single sections of up to three biological replicates for the PD counts.

**Methods S3:** Equation for the calculation of PD densities according to Gunning (1978):

$$\rho=\frac{N}{l}*\left( \frac{1000}{T+1.5*r} \right)$$

ρ: PD density (PD/µm^2^); N: number of PD, l: cell wall length [µm], T: section thickness (~80 nm), r: average PD radius [nm]
